# Microscale Metal Patterning on Any Substrate: Exploring the Potential of Poly(dopamine) Films in High Resolution, High Contrast, Conformal Lithography

**DOI:** 10.1021/acsami.4c07115

**Published:** 2024-11-20

**Authors:** Elliott D. Kunkel, C. Blake Loker, Hunter N. Cowden, Hans D. Robinson

**Affiliations:** Department of Physics, Virginia Tech, Blacksburg, Virginia 24060, United States

**Keywords:** polydopamine, lithography, thin films, photocatalysis, metallization, catecholamines, conformal coatings, photoreduction

## Abstract

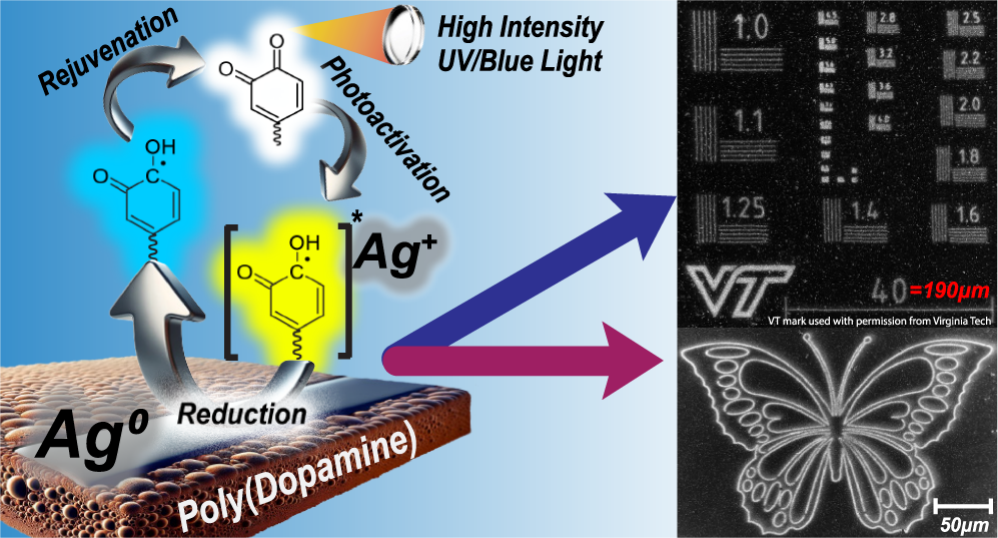

We have explored the potential of poly(dopamine) (PDA) thin films as versatile, high resolution conformal photoresists, using catalytic photoreduction of silver ions to micropattern the film. The combination of photosensitivity, biocompatibilty, and straightforward deposition under mild conditions into thin (∼45 nm) conformal coatings on nearly any material makes PDA films of interest in lithographic patterning on highly nonplanar geometries as well as on soft and biological materials where standard photoresists cannot be used. PDA and poly(norepinephrine) (PNE) films deposited with a standard autoxidation process were investigated along with PDA film deposited with a fast oxidation (FO) technique. Notably, we find that nonspecific deposition of silver off the lithographic pattern is strongly suppressed in PNE and nearly absent in FO-PDA films, which makes very high contrast lithography possible. We attribute this to a lower ratio of catechol to quinone moieties in these films compared to standard PDA films. PNE and FO-PDA films also exhibit smaller silver grain sizes (<40 nm) than standard PDA films, where grains are up to 200 nm in size. We demonstrate laser-scanning lithography patterns at 1.7 μm spatial resolution near the optical resolution limit of the experiment. Continuous silver films can readily be deposited on PDA, PNE, and FO-PDA with blue ( = 473 nm) and UV-A (375 nm) light, but not with green (515 nm) light. The UV light at lower intensities deposits silver several times faster than the blue light but also degrades the deposited silver at high light intensities. Silver films deposited in this way reach the percolation threshold at optical doses (at  = 473 nm) in the range of 10–50 kJ/cm^2^, and SEM images of the films appear nearly pinhole free at comparable doses.

## 1 Introduction

Polydopamine (PDA) is a versatile material originally synthesized to mimic highly adhesive mussel proteins.^1^ Due to a straightforward coating process requiring mild conditions, low cost of reagents, biocompatibility, biodegradability, and ability to conformally coat nearly any surface without the need for a primer, PDA has attracted a sustained interest over the nearly two decades since its discovery.^1−11^ PDA shows great promise in various applications due to its biocompatibility and multifunctional properties. In the medical field, it is suitable for scaffolds, tissue and bone engineering, cancer therapy, photothermal treatments, drug delivery, and regenerative medicine.^6,7,11^ In environmental applications, PDA is being used for sensing organics, metals, and biomolecules, and it excels in water purification of organics, metals, radioactive materials, and salts.^10^ Other notable applications include its use in antifouling coatings, membranes, hydrogels, Li-ion batteries, photosensitive adhesives, solar cells, and as a biomimetic material due to its chemical similarity to eumelanin.^1,5,6,9,10,12−15^

PDA films are also photocatalytically active.^1^ Their highly conjugated structure strongly absorbs both ultraviolet (UV) and visible light, producing mobile charge carriers that can migrate to the film surface, and there induce reactions that can be used for secondary modification of the film as well as more broadly for catalysis.^16^ PDA has also been used for charge transfer in dye-sensitized solar cells, in photodynamic drug therapy, and for enhancing the photoactivity and photostability of CdSe nanocrystals.^16^ PDA films can also act as a photoinitiator for polymer brush growth from the film surface.^12,17^ This was pioneered by Sheng et al. who demonstrated growth of a variety of acrylate- and styrene-based polymer brushes on the film surface, induced by broadband ultraviolet illumination.^12^ Such illumination can also photoreduce ions of noble metals such as silver and gold into metal nanoparticles that adsorb onto the film.^18,19^ Lithographic pattern formation at the mm scale using laser printed photomasks has been demonstrated with this technique.^18^

In this article, we present initial results of microlithographic patterning on PDA and related films that make use of the films’ photocatalytic properties. This is of interest due to an unusual combination of properties in PDA, in addition to its photoreactivity. First, PDA films *can be deposited on nearly any surface*, including a wide array of metals, semiconductors, ceramics, and polymers including fluoropolymers such as PTFE.^5^ PDA also associates strongly with DNA and many proteins and will coat many biomedically relevant surfaces, such as lipid bilayers and polysaccaride film, as well as the outer membrane of living cells.^20^ The deposition usually occurs by immersion in an aqueous bath, but other methods such as spray application are also possible.^21^ Second, the film deposition is self-limiting and therefore produces *conformal films* about 45 nm thick under standard conditions,^1^ even on very intricate and highly curved surfaces, such as tissue scaffolds,^22^ nanoparticles,^3^ nanopores,^23^ and other nanostructured surfaces.^24^ Third, PDA deposition is very mild as it occurs from a dilute aqueous solution at slightly basic pH (>7.5)^5^ and can be applied to a number of biological and other substrates that are not compatible with standard lithographic processes. Taken together, these properties make PDA a candidate photoresist for situations where standard lithography falls short, such as on highly curved or structured substrates, on microparticles, or in biological and other fragile material systems.

Patterning on highly curved surfaces is in demand for applications such as smart textiles,^25^ wearable sensors,^26^ and biomedical devices.^26^ If the curvature or aspect ratio of the substrate is small enough, techniques such as microcontact printing, phase shifting edge lithography, decal transfer lithography, or similar soft lithography techniques are often adequate.^27^ However, the conformal coverage of PDA makes lithography possible on or within substrates with nearly arbitrarily high curvature and aspect ratio, particularly if the substrate is at least partially transparent and can be index-matched to its surroundings. For example, individual fibers in tissue engineering scaffolds^28^ could be patterned at depth with submicron resolution. The same would hold for photonic crystals and other porous photonic structures.^29^ Further, artificial self-assembly, particularly using the so-called patchy particle approach,^30^ requires high precision patterning of microspheres that could be enabled with PDA-based lithography. PDA’s excellent biocompatibility will also allow patterning of delicate biological tissue and structures, possibly down to the level of patterning the outer membrane of individual cells.^3,4,31^

The limited amount of published work on photo patterning PDA films^5,12,18,18−34^ does not yet allow applications such as these to be realized, as demonstrations rarely go beyond patterning at the millimeter scale, nor make any attempt to characterize the quality and mechanism of the patterning process. One exception is the work by Zeng et al.,^18^ who observed that PDA films reduce silver ions through a thermal reaction even when not illuminated, producing nonspecific silver deposition across the entire film that reduces lithographic contrast. (By a “thermal” reaction, we here mean a thermodynamically driven reaction, as opposed to photochemically driven by light absorption.)

Our purpose here is to demonstrate that microscopic resolution is possible in PDA lithography, to measure the photosensitivity of the lithographic process, and to characterize the quality of the lithography, including for photodeposition of high density, nearly continuous metal layers that to our knowledge have not been reported previously. In addition, to validate PDA films for high contrast lithography, we need to show that the nonspecific thermal silver deposition on PDA can be minimized.

Because much of the previous work in this area has examined the photocatalytic deposition of silver on PDA, it is natural to choose this contrast mechanism here as well. Silver is also a convenient choice because it can be deposited in continuous and thick films in comparison to polymer brushes^12^ and is therefore more visible in both optical and electron microscopy. Silver is also suitable for a number of applications. For example, it is an excellent conductor and can therefore be used in electrical interconnects.^35^ Metallic silver is unaffected by hydrofluoric acid and can thus function as an etch masking material.^36^ Silver also has very low optical losses, features strong plasmonic resonances, and therefore is a favored material in a variety of plasmonic devices^31,37,38^ for which ability to directly write submicron silver structures would be valuable. Furthermore, there are many options for modifying the deposited silver to tailor its properties, for instance, through electroless deposition, substitution with a different metal, or modification of surface properties through the application of thiol-bound self-assembled monolayers.^39−41^

The photochemistry of PDA is highly versatile, and our focus on silver patterning should not be taken to diminish the usefulness of other patterning modalities. For instance, patterning with gold^12,19^ may be preferable in biomedical applications to avoid the cytotoxic effects of silver, or when there is a need for a metal pattern that does not oxidize like silver does unless encapsulated. Similarly, a wide variety of polymer brushes can be photochemically grown on PDA, regulating surface properties such as hydrophobicity^12^ and cell adhesion^32^ of the surface. Polymer brushes presenting a range of functionalities can be fabricated,^42^ so that the exposed pattern can be modified as desired with anything from metal nanoparticles^43^ to biomolecules.^44^ The PDA film itself can also be directly functionalized, for instance, with thiols and amines.^1,45^ Photodeposition of metal or an inert polymer brush can therefore enable negative lithography, where the desired surface modification is applied to the unexposed areas of the film.^46^

**Scheme 1:**
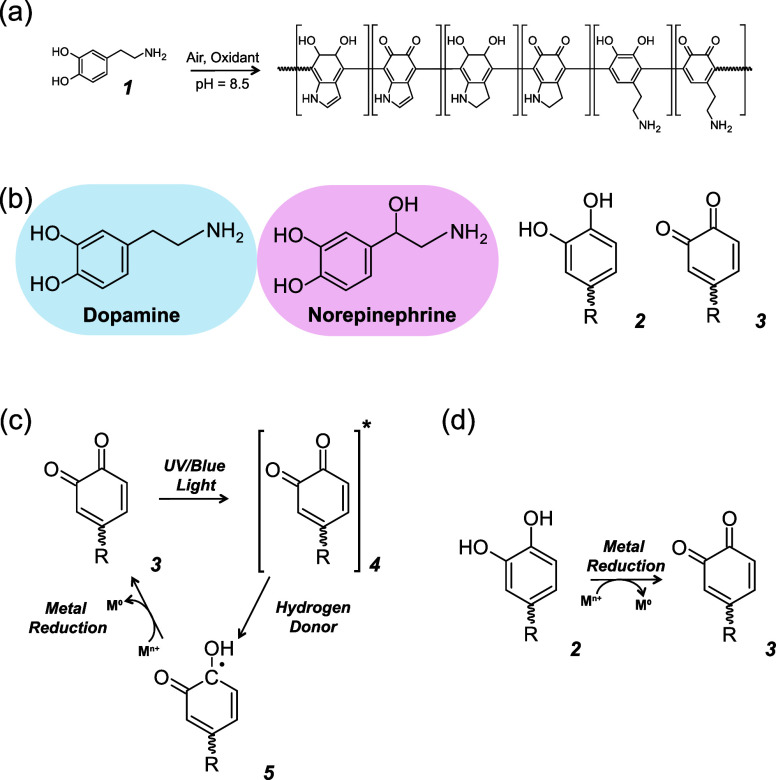
(a) Simplified Depiction of the PDA Polymerization Reaction; (b) Dopamine (DA) **1** and Norepinephrine (NE) Monomers; Catechol **2** and Quinone **3** Moieties; (c) Photocatalytic Reduction of Metal Ion Mediated by **3**; d) Thermal Reduction of Metal Ion Mediated by **3**

The PDA film deposition process is straightforward. The dopamine (DA) monomer is simply dissolved in a basic buffer solution, possibly in the presence of an oxidant, where it undergoes oxidative polymerization that results in a uniform conformal film on all surfaces in contact with the solution, as well as the formation of PDA nano- and microaggregates, some of which become embedded in the film.^3^ The reaction and the resulting polymer structure are both quite complex (See Schemes S1 and S2 in the Supporting Information for a short summary) and not fully understood in every detail.^48^ However, we will mainly focus on the catechol **2** and quinone **3** moieties in the film as they play starring roles in respectively the light-induced and the nonspecific deposition of silver on PDA.^18^ Specifically, the light-induced reduction in Scheme 1c is likely mediated by the quinone groups, in which the absorption of a photon abstracts a hydrogen from solution, forming a ketyl radical, which in turn readily reduces a metal cation while regenerating the original quinone.^18^ The thermal reaction in Scheme 1d is instead caused by catechol groups transforming into quinones while reducing dissolved metal ions.^49^ Even though the latter reaction is slow and self-limiting, because the catechol is consumed in the process, it still leads to a significant reduction in lithographic contrast. A key hypothesis of this study is that due to the nature of the light-induced and nonspecific mechanisms involved in the metal deposition on PDA, a low catechol to quinone ratio (CQR) should inhibit the thermal reduction of silver. Consequently, low CQR films are expected to achieve significantly higher contrast during lithographic patterning compared with high CQR films.

To test this hypothesis, PDA and PDA analogue films with different CQR were prepared and patterned with noncontact laser-scanning immersion lithography (LSIL) at wavelengths from UV-A to the green. Immersion lithography is used because metal deposition requires direct contact between the film and a metal salt solution; it also has the added benefit of improving spatial resolution. With our experimental setup, we are able to produce lithographic patterns of continuous silver films with high uniformity and micron-scale resolution, where nonspecific silver deposition is strongly suppressed or even eliminated in the samples with the lowest CQR values.

## 2 Results and Discussion

### 2.1 Samples

We picked three types of catecholamine films for characterization from the literature. The first type consists of standard PDA films, representing the vast majority of the literature to date, and is formed by autoxidation of the monomer. It deposits at a low rate of approximately 0.02–0.04 nm/min, with the film thickness leveling off at 45 ± 5 nm in 24 h.^12^

The second set of films was deposited with the DA analogue norepinephrine (NE) as the monomer (see Scheme S1 for a summary of the reaction) while keeping all other parameters the same.^50^ The resulting poly(norepinephrine) (PNE) films are believed to be very similar in structure and properties to PDA films, although their surfaces are clearly smoother, with fewer inclusions of polymer aggregates.^51^ Published data also indicate that PNE films likely have a lower CQR than standard PDA.^51^ Though little work has been published on PNE chemistry, a plausible explanation for this is that the benzylic hydroxy group in NE enhances oxidative cleavage reactions during polymerization.^52^

For our third set of films, we added hydrogen peroxide (H_2_O_2_) as an oxidant to the DA solution, catalytically enhancing it with Cu(II) ions in the form of copper sulfate (CuSO_4_). This approach speeds up the film deposition to 0.70–0.80 nm/min, decreases deposition time to less than 30 min, and also results in smoother and more homogeneous films.^49^ Because of the deposition speed, we denote these samples as fast oxidation PDA films (FO-PDA). Significant deposition speed-up can also be obtained with other oxidants,^21−55^ and we would expect these to behave similarly, in particularly because faster oxidation should lead to films with a small CQR, making them prime candidates for testing our contrast hypothesis.^49^

The three films were characterized by XPS for their composition and also patterned with silver via LSIL to characterize lithographic resolution, contrast, and uniformity. The morphology of the resulting silver films was also examined with scanning electron microscopy (SEM). Finally, optical in situ characterization of silver deposition kinetics was performed at varying conditions to obtain the metal deposition contrast curve and information on how it may be optimized. These experiments are each described and analyzed in the following sections.

### 2.2 XPS Analysis

**Figure 1:**
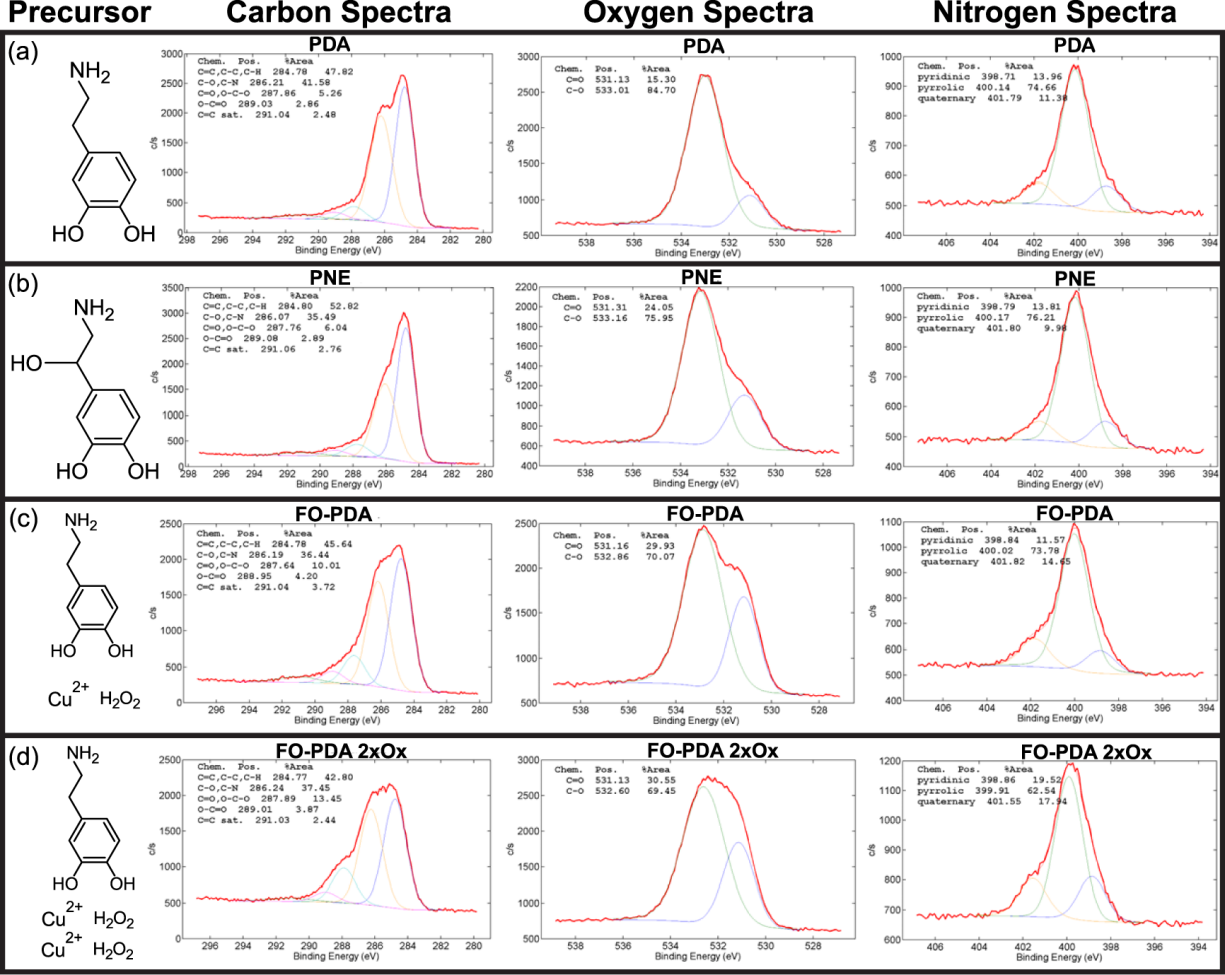
XPS analysis of the 4 different types of catecholamine films. a) PDA made from dopamine using the standard autoxidation method. b) PNE made from norepinpehrine hydrochloride using the standard autoxidation method. c) FO-PDA made from dopamine using the fast oxidation method with a standard concentration of oxidant. d) FO-PDA 2xOx made from dopamine using the fast oxidation method with double the standard concentration of oxidant.

XPS analysis was performed on four different catecholamine films deposited on silicon substrates: PDA, PNE, FO-PDA, and FO-PDA fabricated with twice the standard concentration of oxidant (FO-PDA 2xOx). The PDA and PNE films were deposited for 12 h as outlined in Section 4.2. The FO-PDA and FO-PDA 2xOx were produced as outlined in Section 4.3, except that the FO-PDA 2xOx sample was oxidized with 39.2 mM H_2_O_2_ and 10.2 mM of CuSO_4_. In each sample, spectra were collected for the O 1s, N 1s, C 1s as well as the Cu 2p lines. The XPS spectra are shown in Figure 1, and measured relative atomic concentrations for different oxidation states are shown in Table 1 (Figures S12–S15 contain overall spectra, and Figures S16–S17 contain the Cu 2p spectra for FO-PDA and FO-PDA 2xOx).

**Table 1:** Table of Relative Atomic Percentages of Carbon Species, Oxygen Species, and Nitrogen Species of the Four PDA-like Films (PDA, PNE, FO-PDA, and FO-PDA 2xOx)

Carbon Spectra				
	PDA C 1s(%)	PNE C 1s(%)	FO-PDA C 1s(%)	FO-PDA2xOx C1s(%)
C=C, C–C, C–H	47.82	52.82	45.64	42.80
C–O, C–N	41.58	35.49	36.44	37.45
C=O, O–C–O	5.26	6.04	10.01	13.45
O–C=O	2.86	2.89	4.20	3.87
C=C sat.	2.48	2.76	3.72	2.44

Oxygen Spectra				
	PDA O 1s(%)	PNE O 1s(%)	FO-PDA O1s(%)	FO-PDA2xOx O1s(%)
C=O	15.30	24.05	29.93	30.55
C–O	84.70	75.95	70.07	69.45

Nitrogen Spectra				
	PDA N 1s(%)	PNE N 1s(%)	Fo-PDA N 1s(%)	Fo-PDA2xOx N1s (%)
Pyridinic	13.96	13.81	11.57	19.52
Pyrrolic	74.66	76.21	73.78	62.54
Quaternary	11.38	9.98	14.65	17.94

Copper Spectra				
	PDA Cu 2s(%)	PNE Cu 2s(%)	FO-PDA Cu2s (%)	FO-PDA2xOx Cu2s (%)
Cu(I)	N/A	N/A	36.26	45.62
Cu(II)	N/A	N/A	63.74	54.38

The C 1s spectra consist of five principal peaks: C=C, C–C, and C–H at 284.8 eV, C–O and C–N at 286.2 eV, C=O and O–C–O at 287.6 eV, O–C=O at 289 eV, and saturated C=C at 291 eV. The O 1s spectra contain 2 principal peaks, one corresponding to C–O at 531.1 eV (stemming from catechol groups and to a lesser extent carboxyls and esters) and the other one corresponding to C=O at 533.2 eV (signifying quinone groups and to a lesser extent other carbonyls). The N 1s spectra contain three principal peaks: =NR bonds (here mostly pyridinic amines) at around 398.8 eV, R_2_-NH bonds (largely pyrrolic amines) at roughly 400.1 eV, and R-NH_2_ bonds at around 401.8 eV (also indicating quaternary nitrogen most likely in the form of C-).^5,56,57^

The C–O and C=O peaks in the oxygen spectra are dominated by catechols and quinones, respectively. We can therefore take the ratio  of the areas under the peaks as an indication of the films’ CQR. We obtain , , , and  for the four films. We need to note that in addition to quinones, the PDA reaction also produces some amount of carboxyls (**7**–**12** in Supporting Information Scheme S1 and as reported in literature^58^), as does the PNE reaction (**10**–**13** in Scheme S2 and as seen in literature^52^). The value of CQR in the films is therefore very likely smaller than . The  values are nonetheless sufficiently different that we can draw the qualitative conclusion that . Finally, the similar values of  and  mean that at least as far as quinone production goes, the oxidant is already in excess in the standard protocol and we gain little by adding more to the reaction.

### 2.3 Lithography

**Figure 2:**
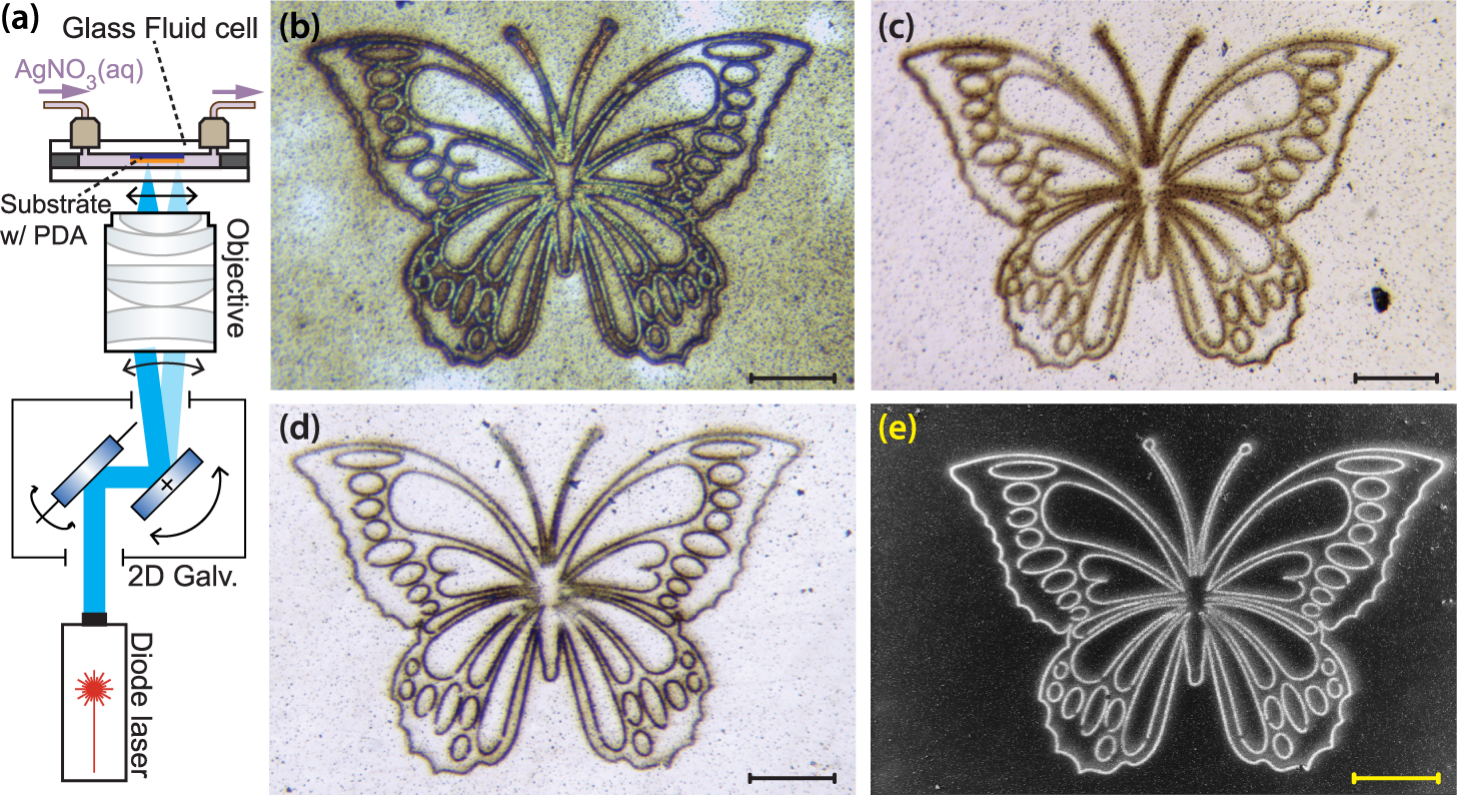
(a) Simplified schematic of LSIL setup that produced the silver patterns in (b)–(e). (b) Micrograph of silver pattern on a PDA film on silicon. (c) PNE film. (d),(e) FO-PDA film, same instance in both images. (b)–(e) are optical brightfield epi-illumination images, and (e) is an SEM image. All scale bars are 50 μm.

A simplified schematic of the LSIL setup is shown in Figure 2a (see Section 4.6 for a full description). A laser beam is first deflected by a 2D galvanometer and then focused on the PDA-coated sample with a microscope objective that transforms the deflection angle into a position on the sample. The sample is mounted inside a glass microfluidic cell so that a deoxygenated 50 mM solution of AgNO_3_ can flow across the sample, and from which the silver metal is deposited.

Optical micrographs of laser-patterned metallic silver deposited on PDA, PNE, and FO-PDA films on silicon are shown in Figure 2b–d. The patterns were produced with a microscope objective (Olympus Plan N, 10×/0.25 NA) using blue light with a wavelength of 473 nm at a total power of 12 mW, and scanned at a speed of approximately 10 mm s^–1^. The total line dose to produce the images was approximately 0.49 J mm^–1^. Under these conditions, the deposited silver is initially nearly transparent but appears brown or black in the optical image after a few days of exposure to air, which converts some of the silver particles to strongly light-absorbing silver oxide (see Supporting Information Figure S2). The randomly distributed dark spots that can also be seen in the micrographs are not silver oxide, but polymer aggregates that form in solution and incorporate in the film during deposition.^3,12,21,51,59^ Fewer such particles incorporate in the PNE^51^ and FO-PDA^49^ films, which is part of the reason these films appear smoother. As a comparison, Figure 2d shows an SEM micrograph of the same FO-PDA pattern as that in Figure 2e. The silver here appears white, as the signal was collected with an in-lens detector that measures SE1 electrons (produced during the electron beam’s entry into the sample) that generate a signal that is sensitive to both surface topography and average atomic number at the beam focus.

#### 2.3.1 Lithographic Resolution

**Figure 3:**
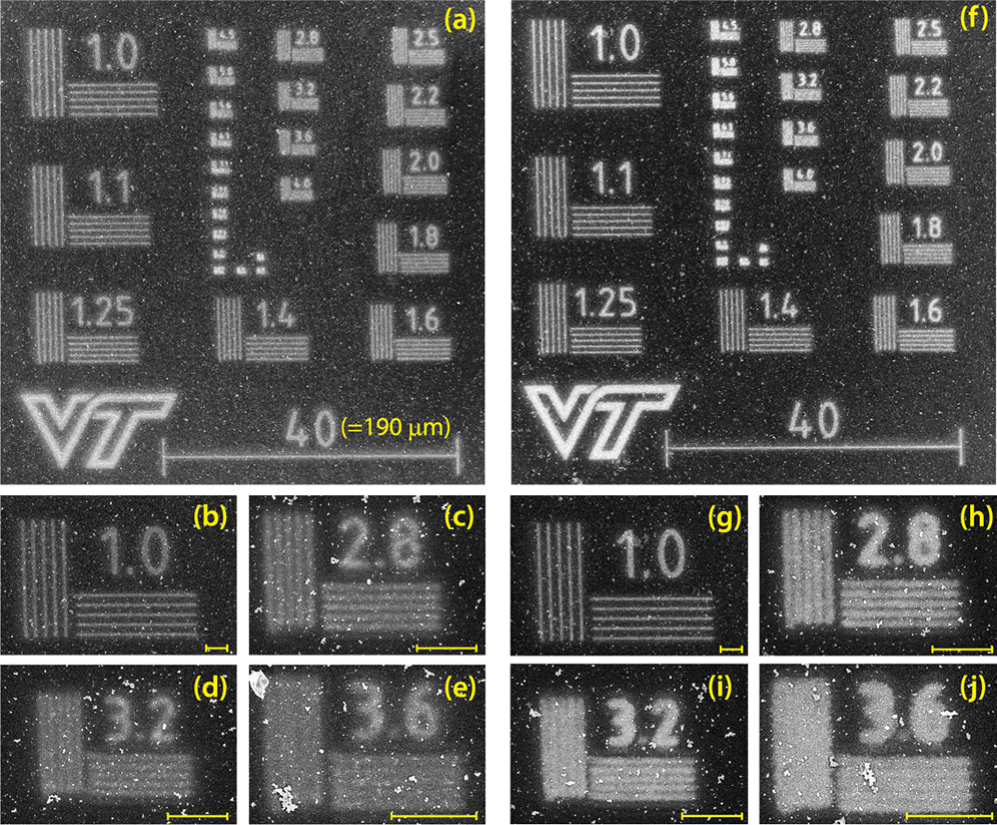
ISO #2 test pattern in metallic silver produced on PDA films on Si. Exposure was performed through laser scanning with 473 nm light at a 0.26 J/mm line dose in (a)–(e), and at 0.52 J/mm in (f)–(j). The scan speed was 5 mm/s, and the optical power at the substrate was 13 mW. The scale bar marked “40” is 190 μm long on the substrate. All other scale bars are 10 μm. (a) and (f) Overview of patterns. The numbers at each sub pattern indicate resolution in line pairs (lp) per unit, where 1 unit = 4.8 μm. (b) and (g) Subpatterns with 4.8 μm resolution. (c) and (h) 1.7 μm resolution. (d) and (i) 1.5 μm resolution. (e) and (j) 1.3 μm resolution. VT mark used with permission from Virginia Tech.

To determine the spatial resolution of the lithographic pattern, we fabricated ISO #2 test patterns (ISO standard 3334:2006) deposited under conditions close to those in Figure 2. The patterns in the SEM micrographs in Figure 3a–e were deposited on PDA with a line dose of 0.26 J/mm, while those in Figure 3f–j received twice as much light (0.52 J/mm). The scale bar at the bottom of Figure 3a defines the length unit of the pattern, and the number within each subpattern indicates the density of the associated lines in line pairs (lp) per length unit. Here, the 40 unit bar is 190 μm long. Accordingly, the 1.0, 2.8, 3.2, and 3.6 lp/unit subpatterns correspond to 4.8, 1.7, 1.5, and 1.3 μm resolution, respectively. The lines remain resolvable at least to the level of the 2.8 lp/unit subpattern, corresponding to 590 lines/mm or a resolution of 1.7 μm (see Figures S3–S8 for quantitative analysis). This should be compared to the objective’s manufacturer specified resolution of 1.3 μm. Given that lithography was performed through approximately 0.5 mm of water, for which the objective was not aberration-corrected, as well as a slight astigmatism in the experiment, we conclude that the lithographic resolution in this case is limited by the optics rather than the PDA film itself.

**Figure 4:**
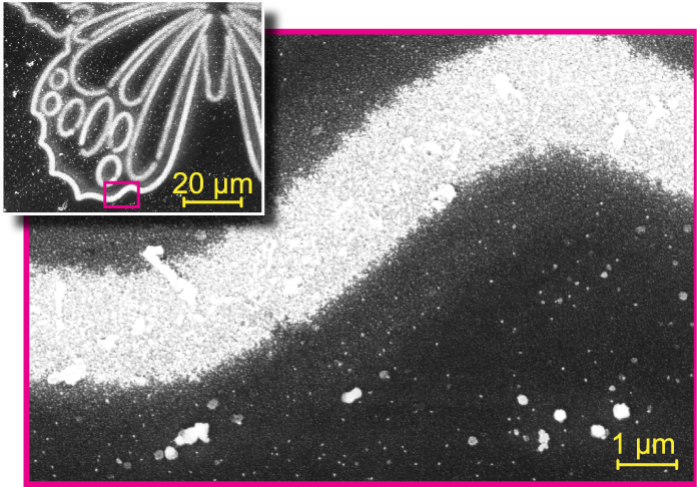
SEM micrograph of a pattern exposed at high dose (*D* = 0.99 J mm^–1^), producing a sharp line with minimal gray tones.

In most kinds of lithography, high quality patterns are stencil-like with as abrupt of a transition as possible at the pattern’s edge. We can achieve this effect by simply overexposing the patterns, as shown in Figure 4, but this comes at the cost of a reduction in spatial resolution. The resolution can be maintained if the resist contrast curve is nonlinear, so that very little deposition occurs at low doses, followed by rapid increase in deposition to continuous metal coverage over a relatively narrow range of doses. Standard photoresists have this property,^60^ and we discuss ways this could be achieved in PDA in Section 2.5.

#### 2.3.2 Silver Film Morphology and Thermal Silver Deposition

**Figure 5:**
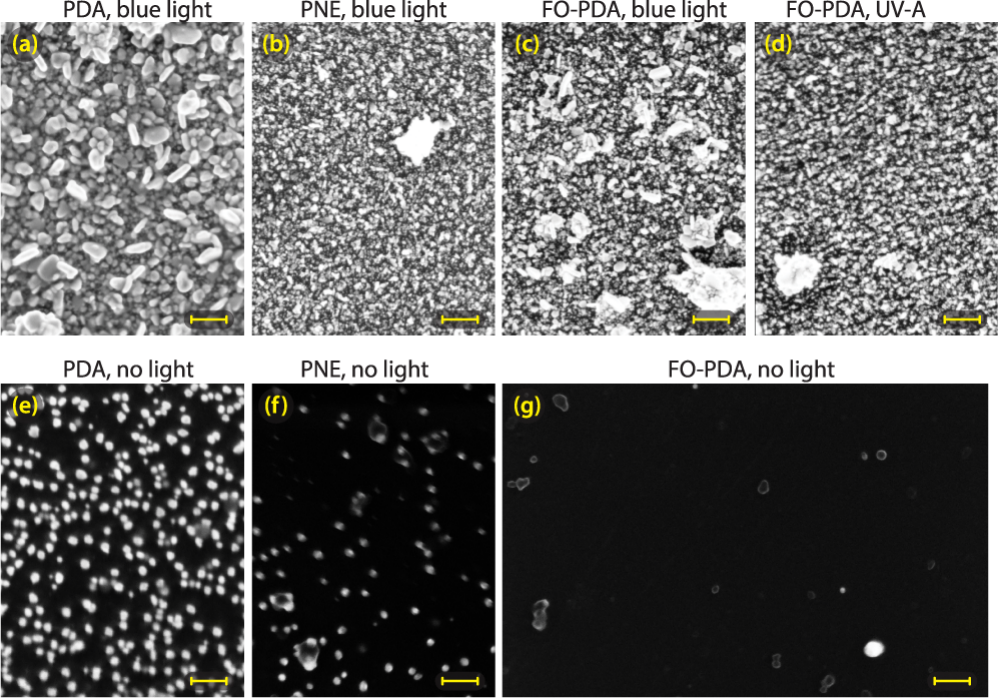
High resolution SEM micrographs of silver deposition on (a) PDA film, blue light dose = 32 kJ/cm^2^, (b) PNE film, blue light dose = 58 kJ/cm^2^, (c) FO-PDA film, blue light dose = 32 kJ/cm^2^, (d) FO-PDA film, UV-A light dose = 200 kJ/cm^2^, (e) PDA film, 1 h immersion in AgNO_3_ solution with no light, (f) PNE film, 1 h immersion with no light, and (g) FO-PDA film, 1 h immersion with no light. All scalebars are 200 nm.

High resolution SEM imaging, shown in Figure 5, demonstrates that the silver morphology differs among the three types of films. All silver coatings contain a range of grain shapes and sizes, but the largest variation is seen in the PDA film, Figure 5a, where many of the grains are elongated and can reach 200 nm in size. In the PNE and FO-PDA films (Figure 5b,c), by contrast, grains are more compact and generally measure no more than ∼40 nm.

**Figure 6:**
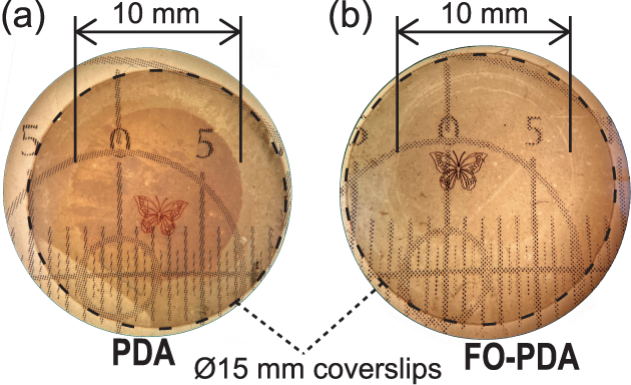
Silver patterns on 15 mm diameter coverslips coated with (a) a PDA film and (b) an FO-PDA film. As indicated, only the central 10 mm of the coverslip was exposed to the AgNO_3_ solution, as the periphery was protected by a gasket. The subdivisions of the printed scales visible through the substrates are in mm.

In addition to the light-induced silver deposition, metal is also deposited in areas that were not illuminated by light. This can be clearly seen by the naked eye in Figure 6a, where silver has been patterned on a PDA-coated flow cell window at the millimeter scale. In the figure, the entirety of the substrate, a 15 mm diameter round glass coverslip, is shown. The substrate is clearly darker in the central 10 mm section, which was exposed to the AgNO_3_ solution, compared to the perimeter, which was covered by a gasket and thus protected from the solution. By contrast, when we perform the same experiment on FO-PDA, which has a lower CQR, the darkening of the central area is much less prominent (Figure 6b).

We can also observe the effect on a microscopic level in the SEM micrographs in Figure 5e,g, which depict areas on PDA, PNE, and FO-PDA samples that have been immersed in a 50 mM AgNO_3_ solution for 1 h while kept in the dark. The silver particles are the compact white shapes, while the darker, less defined shapes are polymer aggregates. A considerable number of silver nanoparticles are evident on the PDA film, with an approximate tally revealing around 327 particles in the captured image. The PNE micrograph presents a significantly lower number of smaller particles, roughly 81 in number, whereas the FO-PDA film barely features any, with only 3 or 4 particles discernible. Translating these observations into particle density yields 132 particles/μm^2^, 33 particles/μm^2^, and fewer than 1 particle/μm^2^ for the PDA, PNE, and FO-PDA films, respectively. These values are consistent with the qualitative estimates of CQR obtained from XPS data and thus corroborate our contrast hypothesis. However, there can be substantial sample-to-sample and spot-to-spot variations, so these numbers should only be taken as indicative estimates of film differences. Optical measurements covering a wider area are detailed in Section 2.4.1.

### 2.4 Optical Measurement of Deposition Kinetics

We performed in situ optical measurements of light-induced silver deposition on PDA, PNE, and FO-PDA films at wavelengths of 375 (UV-A), 473 (blue), and 515 nm (green). The silver deposition rate was estimated from the transmission of nonphotoreactive 632 nm red light through the polymer-coated substrate during film growth (see Section 4.6 for the experimental setup).

When silver nanoparticles develop on the surface, they strongly interact with red light. At this stage, the silver growth is in island mode, where single nanoparticles are deposited randomly across the surface, initially avoiding mutual contact. SEM imaging (Figure 5e–g) also indicates that nanoparticles produced at the beginning of the deposition process are less than 40 nm in diameter in all studied film types, including on PDA, even though silver films on PDA contain much larger grains at later stages (see Figure S18 as well as Figure S2 in ref (18)). In this size range, silver nanoparticles scatter light very weakly, so that the dominant source of attenuation of the red light is absorption, which in turn is proportional to the volume of the particles, and thus to the amount of deposited silver.^61^ Therefore, at low to moderate particle densities, well below the formation of a continuous film, the light extinction coefficient *E* will be proportional to the amount of deposited silver, and the process will obey a 2D version of the Beer–Lambert law:
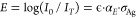
1where *I*_0_ is the light intensity incident on the sample, *I*_T_ is the intensity transmitted through sample,  is the surface density of silver, ϵ is the optical absorptivity of the silver, and α_E_ is proportional to the average extinction (absorption + scattering) cross section for the silver nanoparticles. We can therefore use *E* to determine the relative deposition rate of silver, at least during the initial stage of metal deposition.

**Figure 7:**
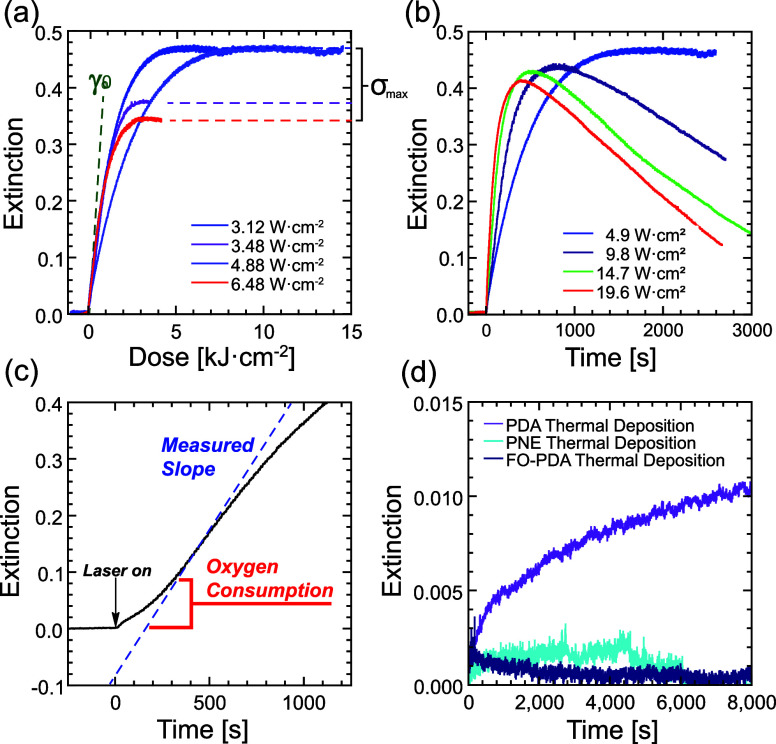
(a) Representative traces of optical extinction (contrast curves) during UV-A illumination of FO-PDA. After the depositing laser is switched on, silver deposition is initiated at rate γ_0_ and then asymptotically approaches a maximum surface density . (b) The film is gradually degraded by exposure to high intensity UV-A light, which leads to silver loss and film destruction during long duration exposures. (c) When some residual oxygen remains in solution, silver deposition sets on gradually, increasing until oxygen adjacent to the film has been consumed. (d) Comparison of thermal silver deposition in the dark for the different catecholamine films.

Typical traces of *E* vs time are shown in Figure 7a for FO-PDA samples exposed to UV-A light at different intensities. As *E* is proportional to , these traces are the contrast curves of the lithography, where the slope  is proportional to the silver deposition rate, at least during the early stages of the deposition. We expect γ to be at its maximum when the light is turned on at time *t* = 0 and then gradually decrease as consumption of reactive species or steric hindrance from already deposited silver slows down the deposition rate, until the silver density asymptotically approaches its maximum value . However, the initial value of γ, denoted γ_0_, would not depend on these long-term factors and therefore measures the intrinsic silver photoreduction rate in the film.

In films exposed to UV light of sufficiently high intensity (above 8 W/cm^2^), we also observe a degradation of the film manifesting as a decrease in silver coverage at long times, as is evident in Figure 7b. This degradation is not visible at lower intensities of UV-A light, nor with blue light exposure at any intensity we used. Since we can avoid or delay the damage by illuminating the film with light at sufficiently low intensities, this effect should not be an impediment to lithography, at least in the UV-A range.

Contrary to our expectation of γ taking on its maximum value at *t* = 0, we sometimes observe that the onset of deposition occurs gradually, with maximum γ obtaining up to several tens of seconds after the laser is turned on, as seen in Figure 7c. We attribute this behavior to the consumption of residual oxygen due to imperfect dexoxygenation of the AgNO_3_ solution. Although this effect is usually small, it does produce some variability in the initial value of . However, since oxygen near the surface is consumed comparatively rapidly, γ changes very little in the time it takes for oxygen to disappear. We therefore take the maximum value of  as a reasonably reliable measure of γ_0_ in the small number of cases where this effect is seen.

#### 2.4.1 Thermal Silver Deposition

The nonspecific silver deposition on the samples was also characterized with the optical setup. The red laser beam was now expanded to approximately 6 mm in diameter so that variations in deposition rate across each sample were averaged out. The shorter wavelength lasers were kept off, while red light transmission was measured over more than 2 h. The results are plotted in Figure 7d. Deposition is clearly visible in the PDA sample, although the deposition rate is at least 2 orders of magnitude smaller than in a typical light exposure, and  is also much smaller, likely because of consumption of the limited amount of catechols available for metal reduction in the dark. By contrast, any silver deposition in PNE and FO-PDA films is below the detection limit of the experiment. This is in line with the microscopic observations in Figure 5, except that the optical measurement cannot distinguish the thermal deposition rates in PNE and FO-PDA.

#### 2.4.2 Light-Induced Ag Deposition Kinetics

**Figure 8:**
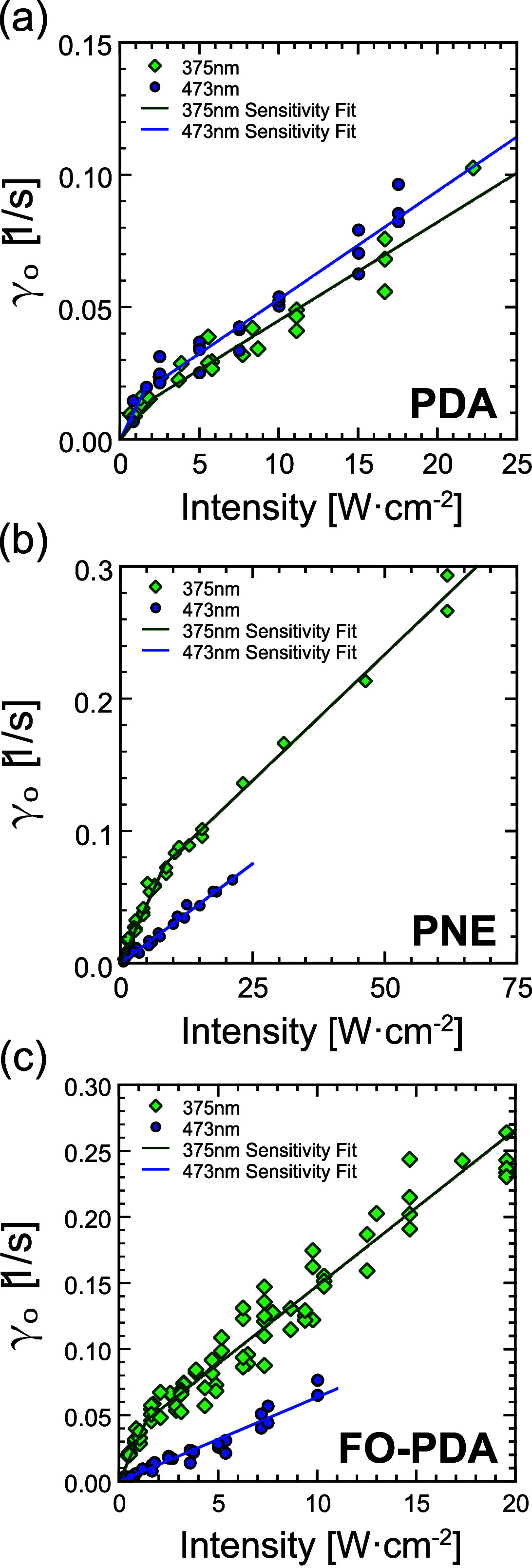
Optically detected intrinsic silver deposition rate γ_0_ as a function of incident light intensity *I*_dep_ on glass coated with (a) PDA, (b) PNE, and (c) FO-PDA.

To characterize the sensitivity of the light-induced deposition in different films, we measured γ_0_ with focused laser light at UV-A (λ = 375 nm) and blue (λ = 473 nm) wavelengths as a function of light intensity. Green light (λ = 515 nm) did not produce any measurable silver deposition, even at the highest intensities (See Figure S1). At each combination of wavelength, light intensity, and film type, we collected up to 5 traces like those in Figure 7a. The values of γ_0_ obtained from these measurements are plotted vs light intensity in Figure 8.

At low UV light intensity (<2.6 W/cm^2^ for PDA, < 8.1 W/cm^2^ for PNE, and <1.6 W/cm^2^ for FO-PDA), there is a clear linear relationship between the deposition rate and light intensity, , consistent with first-order kinetics for the light-induced reduction, that is, a deposition process where one absorbed photon with some probability produces a single radical in the film that then goes on to reduce one silver atom. The coefficient  is the silver reduction reactivity constant of the film for a given wavelength λ and concentration of silver ions , or equivalently, the optical sensitivity for silver deposition in the film. At higher light intensities, γ_0_ increases sublinearly with light intensity in the PDA film illuminated with blue light, and in all films illuminated with UV-A. The slope of the curve in the sublinear range is roughly constant, so that we can define a differential sensitivity  at high powers. The difference between *A* and *A*′ may be due to a saturation phenomenon in either light absorption or silver ion diffusion, or possibly to damage to the film at higher light intensities. The values of *A* and *A*′ are determined by linear fits to the data. These values, as well as the crossover intensity *I*_*xo*_ from linear to sublinear behavior are summarized in Table 2.

**Table 2:** Ag Deposition Sensitivities *A*, *A*^′^, and Crossover Intensity *I*_*xo*_ for PDA, PNE, and FO-PDA Films

	λ = 375 nm (UV-A)			λ = 473 nm (Blue)		
	*A*[cm^2^/kJ]	*A*′[cm^2^/kJ]	*I*_*xo*_[W/cm^2^]	*A*[cm^2^/kJ]	*A*^′^[cm^2^/kJ]	*I*_*xo*_[W/cm^2^]
PDA	9.6 ± 0.9	2.6 ± 0.6	2.58	12.1 ± 1.9	4.3 ± 0.7	2.50
PNE	9.5 ± 0.3	3.9 ± 0.4	8.13	3.0 ± 0.6	–	–
FO-PDA	32.0 ± 1.2	4.1 ± 0.2	1.61	6.3 ± 0.2	–	–

For both PNE and FO-PDA films, the deposition rate at low light intensity is 3–5 times higher with UV-A light compared to blue light, while the UV advantage in sensitivity largely disappears at higher light intensities. We also know that high intensity UV light gradually degrades the film. In other words, if the lithography light source is moderately bright, it is advantageous to use a shorter UV wavelength to which the film is more sensitive, which also enables higher resolution lithography. At high light intensity, it is better to use a longer blue wavelength.

#### 2.4.3 Percolation Threshold

Though we are not directly measuring the amount of deposited silver in this paper, optical measurements and micrographs can provide qualitative information about when the silver film is approaching full coverage. This is possible because as more and more silver particles crowd onto the substrate, they will eventually begin to touch each other. The film then evolves from a nonconducting layer of isolated islands toward a fully connected metallic film. At some density, conduction electrons gain free movement across the film in response to incident light, producing a spectrally flat reflectivity characteristic of bulk metal rather than the strongly resonant response of isolated metal nanostructures. This transition is known as the *optical percolation threshold* and is straightforward to observe optically.

**Figure 9:**
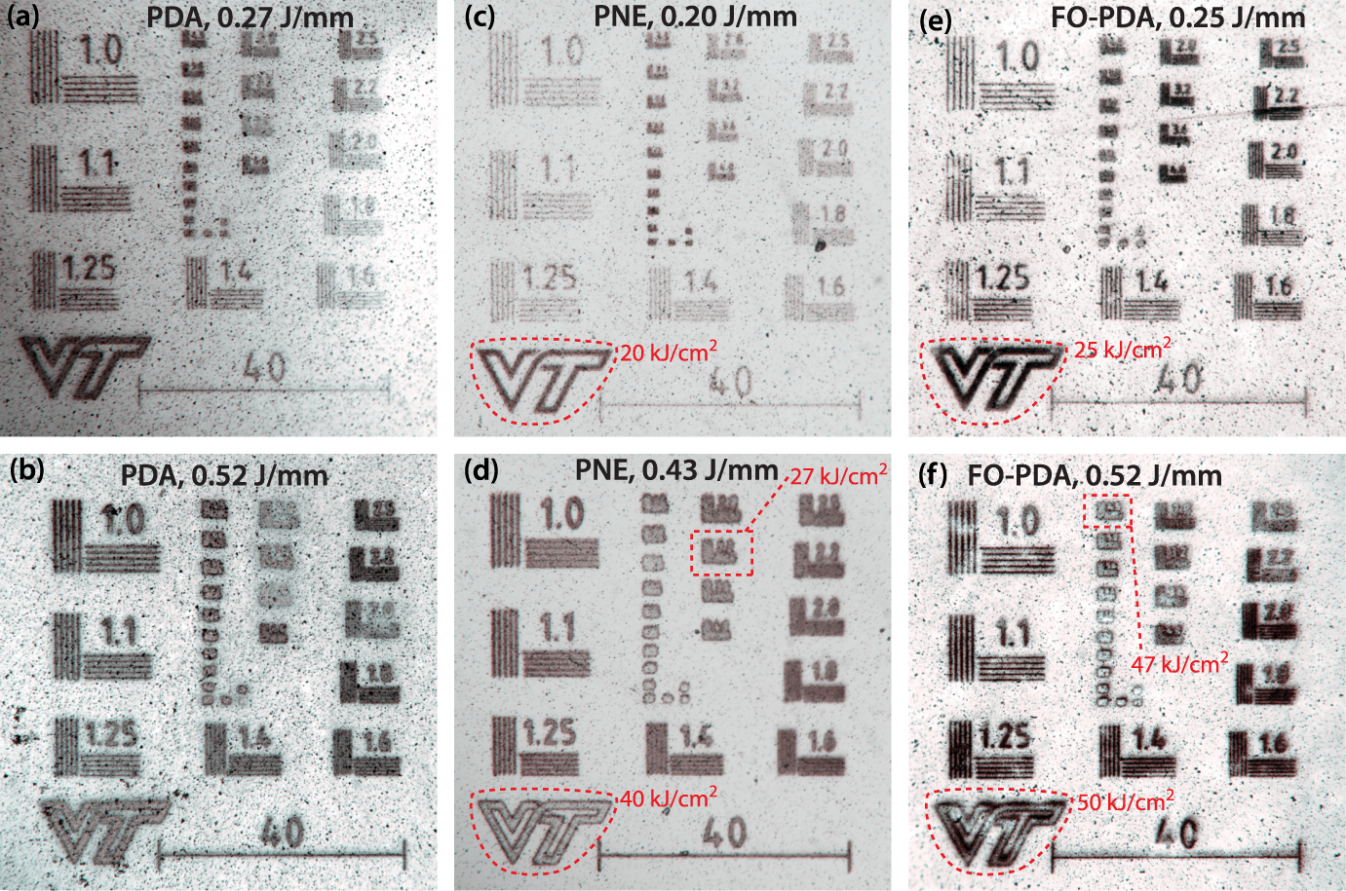
Optical micrographs of 18 day old resolution test patterns deposited with λ = 473 nm light on PDA, PNE, and FO-PDA as indicated in the figure. Each pattern was deposited with the indicated line dose so that varying line densities in each subpattern (shown in linepairs/unit (lp/unit), where one unit is 4.75 μm) produces different area doses. Examples of area doses are indicated in red. The line density for the “VT” logo is 990 lp/mm. VT mark used with permission from Virginia Tech.

This is clear from the optical micrographs of the test patterns shown in Figure 9, where the appearance of the silver changes from black/brown to metallic gray when a given light dose is exceeded, and the percolation threshold is reached. This reflectivity differs enough from the reflectivity from the silicon substrate that it can be distinguished even in freshly fabricated samples (see Figure S2), but the presence of black silver oxide particles creates a significantly clearer outline in the micrographs.

Achieving percolation is a required step toward depositing a fully continuous, pinhole-free film, which is needed in cases where the metal layer is intended as a mask for processes such as etching or ion implantation. We will therefore use the dose *D*_perc_ at the percolation transition as a convenient, although rough, indicator of a fully developed silver pattern that provides nearly complete coverage of the underlying substrate.

The test patterns contain lines that are drawn at a specific density in each subpattern, and because they are drawn with a constant line dose, each subpattern corresponds to an area dose proportional to the line density (See Discussion S1–S2 and eqs S1–S6 for more details.) In PDA, the percolation transition appears to be very gradual compared to the PNE and FO-PDA films, possibly because of the larger grain sizes in the PDA film, which makes it difficult to visually pinpoint a transition point. This is more straightforward in PNE and FO-PDA, and we see that the PNE sample reaches percolation when irradiated with a blue light dose *D*_perc_ between 27 kJ/cm^2^ and 40 kJ/cm^2^, while a larger dose, on the order of 50 kJ/cm^2^, is required for the FO-PDA sample. It is worth noting that the samples depicted in Figure 5 and that on visual inspection appear as nearly pinhole-free films of continuous silver, were produced under very similar conditions as the samples in Figure 9 and at doses comparable to *D*_perc_.

**Figure 10:**
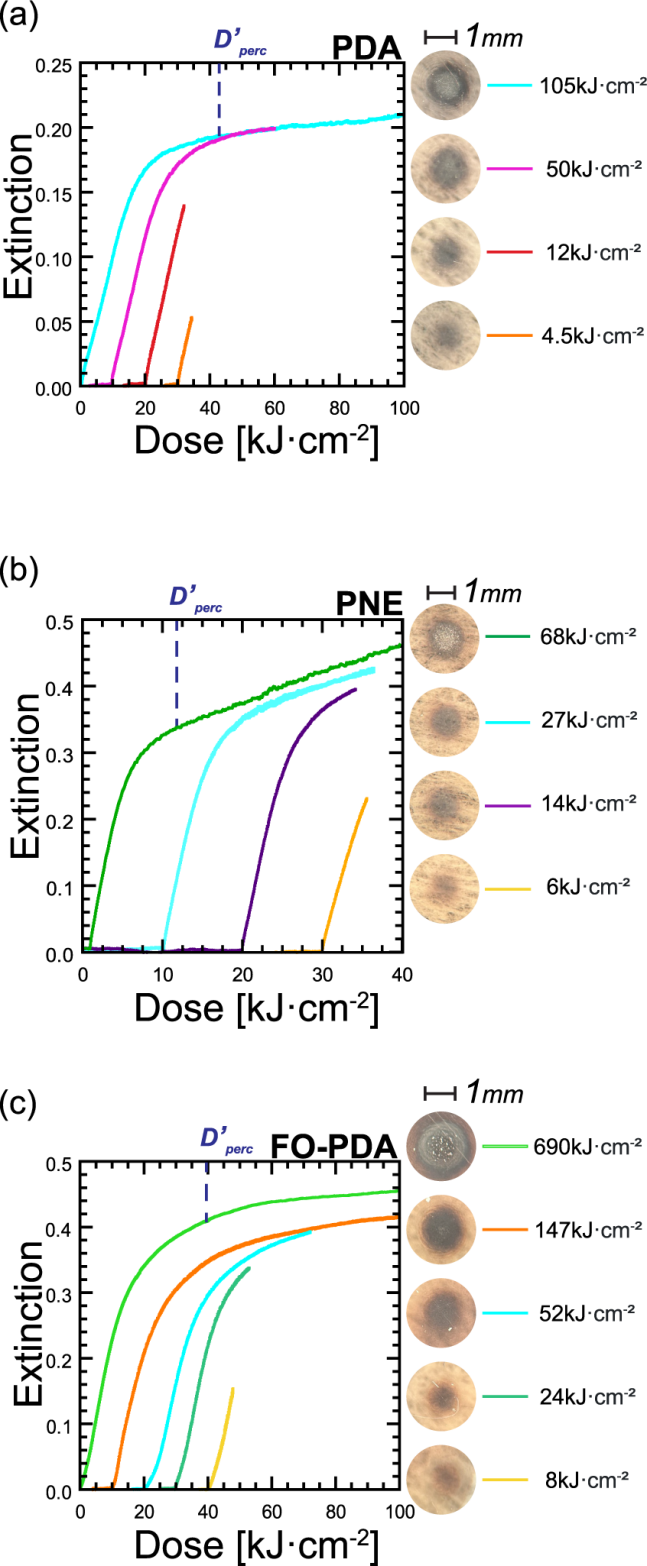
Optical extinction during Ag deposition with blue (λ = 473 nm) light with a 13 W/cm^2^ intensity focused into a 1.3 mm diameter spot on the sample. Deposition was carried out to different target doses, and spots were imaged as shown. (a) Deposition on a PDA film. (b) Deposition on PNE. (c) Deposition on FO-PDA. Traces in all panels are offset horizontally in 10 kJ/cm^2^ increments for clarity.

The percolation threshold can alternatively be measured with the optical deposition setup, which is illustrated in Figure 10. The contrast curves in the figure can be clearly divided into two regions: a low dose region with high γ, and a high dose region with much lower γ values. The transition between the regions is too abrupt for this to be caused solely by steric hindrance by the previously deposited silver. More likely, the transition marks the first stages of the percolation transition, where individual silver nanoparticles begin to merge into larger clusters, producing a different optical response of the film with increased silver deposition. If this is what occurs, we expect the film color to change to metallic at somewhat higher doses. Figure 10 plots multiple traces, interrupted at various points along the contrast curve along with optical images of the resulting silver spots on the film. In the case of the PDA film, a very clear metallic patch forms at *D* = 50 kJ/cm^2^, while the transition region ends at 20 kJ/cm^2^. For the other two films, this is more difficult to see, but on close examination of the images it is apparent that metallization in both PNE and FO-PDA begins at doses within and slightly above the transition region. Therefore, we designate the end of the transition region as , with the prime signifying that this dose represents a different estimate of the percolation threshold than *D*_perc_. In the three films, we obtain  = 16 kJ/cm^2^,  = 8 kJ/cm^2^, and  = 20 kJ/cm^2^ (see Figures S9–S11). These values are up to  times smaller to the estimates from Figure 9, which is satisfactory given the difference of estimation method, and in particular the much tighter focus in the microscopically patterned samples that results in a difference in laser intensity at the surface of nearly 5 orders of magnitude between the two measurements: 13 W/cm^2^ in Figure 10 versus 0.54 MW/cm^2^ in Figure 9.

#### 2.4.4 Lithographic Uniformity

The sharpness of the percolation transition can be used to qualitatively determine the uniformity of the lithographic pattern. In particular, the similarity of grain morphology in silver films deposited on PNE and FO-PDA makes a comparison between these films straightforward. From Figure 9, it is then clear that silver patterns on PNE are significantly more uniform than on FO-PDA. The larger grain size and a more gradual percolation transition in PDA films makes comparison difficult, but a close examination of for instance Figure 9b makes it clear that the PNE film uniformity is superior to that in PDA films as well. The scatter in the values of γ_0_ around the lines of best fit in Figure 8 confirm this result, as the variations are much greater in the plot of the FO-PDA deposition rates than in the corresponding plot for PNE, with standard PDA occupying a middle ground. Comparing the widths of the transition regions in the plots in Figure 10 leads to the same conclusion.

### 2.5 Summary of Results and Future Work

The experimental results (except those already listed in Table 2) are summarized in Table 3. We have found that all three film types are able to support lithography at a spatial resolution of better than 1.7 μm, which here is limited by the optical setup rather than the films. In fact, the thickness of the films, the small size of the deposited silver particles and the sharpness of the line boundary in Figure 4 indicate that a substantially better resolution should be feasible, particularly in PNE and FO-PDA.

**Table 3:** Summary of Lithographic Figures of Merit and Other Relevant Results for the Three Studied Film Types

	Lithography			Film Properties				Film Dep. Rate	
Film	Spatial Res.	Contrast	Uniformity	Smoothness	Grain size		Inferred CQR	*D*_pers_	
PDA	1.7 μm	Poor	Mod.-Good	Poor	200 nm	5.54	High	N/A	16
PNE	1.7 μm	Good	Very Good	Moderate	40 nm	3.16	Low	27–40	8
FO-PDA	1.7 μm	Very Good	Moderate	Moderate	40 nm	2.34	Very Low	∼50	20

Our second figure of merit is contrast, which here is limited by nonspecific deposition of silver. On this score, PDA performs rather poorly, while PNE has a much reduced nonspecific deposition rate. However, FO-PDA is clearly the superior choice, with little to no nonspecific deposition observed. These results track well with the CQR in film as estimated from  ratio obtained from XPS data.

When it comes to pattern uniformity, as estimated by three separate measures, PNE is clearly the best performer with FO-PDA exhibiting substantially greater variation. The morphology of the silver film on PDA makes comparison with FO-PDA more uncertain, but we are nonetheless able to conclude that its pattern uniformity is not as good as that in PNE.

Although the roughness in all films is substantial and likely too high for many lithographic applications, we have not attempted to improve film smoothness in this work. However, there are several approaches that improve PDA film smoothness and that could ameliorate this problem, such as adjusting monomer concentration,^56^ deposition time and pH,^62^ or oxygen concentration,^63^ as well as postdeposition annealing^64^ or sonication.^65^

A limiting factor for PDA films’ use in lithography is that they are very slow, requiring doses of at least several kJ/cm^2^ in the mode we have used them here. This should be compared with standard photoresists, where the required exposure is some 5 orders of magnitude smaller, in the range of tens to a few hundred mJ/cm^2^. This difference stems directly from the chemical amplification used in commercial photoresists, where the absorption of a single photon releases a photoacid that ends up modifying the solubility of a large number of polymer side groups.^60^ One can imagine a similar amplification scheme here in which samples are first exposed to a low optical dose, producing a low density of silver seed particles on the exposed areas of the substrate. The silver seeds can then be grown into a continuous metal layer through electroless plating.^39,66^ This technique is reminiscent of silver halide-based photography^67^ and has in fact already been demonstrated in PDA films.^68^ Therefore, it is likely also applicable to lithographic applications.

The response of standard photoresists is nonlinear at low doses, which makes it possible to combine high spatial resolution with sharply defined, gray scale-free lines. Unfortunately, the contrast curves in Figure 7a and Figure 10 are linear at low doses. The one exception to this is the contrast curve in Figure 7c where the likely presence of oxygen during deposition produces a nonlinearity of the desired type. This effect could possibly also be achieved by binding a consumable inhibitor to the surface instead of relying on difficult-to-regulate low oxygen concentrations. Another option for increased contrast could be to apply careful etching of the silver after or along with the photodeposition so that no metal remains on the surface except where the silver deposition density exceeds the etched amount of metal.

## 3 Conclusions

We have carried out a preliminary exploratory investigation of the lithographic potential of PDA, PNE, and FO-PDA films, motivated by the combination of the versatile photocatalytic properties of these films and their ability to readily form thin conformal coatings on nearly any surface as well as by their biocompatibility and mild deposition conditions. We have clearly shown that high resolution lithography through optically induced silver reduction is possible on these films and potentially other catecholamine films. We have demonstrated 1.7 μm resolution, limited by the optics, but the achievable resolution is likely substantially better. In fact, the Rayleigh resolution criterion () indicates that with the blue λ = 473 nm light, and optics with N.A = 1.15 (achievable for an aqueous immersion objective), a resolution of 250 nm should be within reach. Even higher resolutions would be possible with light of shorter wavelengths. However, we have also found that while UV-A light causes silver deposition with even greater efficiency than blue light, it also damages the films at sufficiently high intensities. This damage may prove to be what limits the lithographic resolution in this system.

A main result of this work is that nonspecific deposition of metal can be suppressed in areas that are not exposed to light, enabling high contrast lithography. Within the sensitivity of our experiment, we observe little or no silver deposition in rapid oxidation FO-PDA films and only a small amount in PNE films. We attribute this suppression to a substantial reduction in the catechol content in the PNE and FO-PDA films.

In summary, the results of this initial exploration are encouraging. High resolution patterning of good uniformity is clearly possible, and very high lithographic contrast can be obtained in rapidly oxidized films, such as our FO-PDA samples. There are also clear avenues to pursue that could mitigate the obstacles and limitations that we have identified. Therefore, it appears that with further work PDA-based films may be able to find a role as a form of high-contrast microlithographic resists, particularly in applications where traditional photoresists prove challenging or impossible to use, such as on strongly curved or deeply structured substrates, soft or biological samples, or in other situations where low toxicity or mild deposition conditions are required.

## 4 Experimental Section

### 4.1 Substrate Preparation

All substrates were cleaned in a solution of 1 part v/v of 30% hydrogen peroxide slowly mixed with 3 parts concentrated sulfuric acid in a pyrex beaker (a “piranha clean”). The substrates were placed in a custom-machined PTFE holder, immersed in the freshly prepared piranha clean for 10–15 min, and then rinsed with copious amounts of DI water. Residual water was removed from the substrates with a stream of dry air. WARNING: Both liquid and vapor phases of the piranha clean are extremely corrosive to the skin, eyes, and respiratory tract, and appropriate personal protective equipment must be worn at all times when preparing and handling the solution. Waste must be discarded into a glass bottle with a vented cap.

### 4.2 PDA/PNE Film Synthesis

99% Dopamine hydrochloride from Sigma-Aldrich or 99% 3-Hydroxytyramine hydrochloride (norepinephrine) from Acros Organics was removed from refrigeration and allowed to equilibrate at ambient temperature for at least 20 min. A 100 mL solution of 50 mM Trizma (Tris) buffer was then prepared with DI water in a 250 mL beaker, after which the pH was adjusted to 8.50 ± 0.02 with 1 M HCl and NaOH solutions. Substrates previously washed in a 3:1 piranha clean were fully submerged in the buffer. 200 mg (2 mg mL^–1^) of DA was dissolved in the buffer under vigorous stirring. The solution was then incubated under moderate stirring for 12–16 h. Finally, the samples were washed with DI water, dried with a stream of pressurized dry air, and stored for up to 10 days in the dark until used.

### 4.3 FO-PDA Film Synthesis

99% Dopamine hydrochloride (DA) from Sigma-Aldrich was removed from refrigeration and allowed to equilibrate at ambient temperature for at least 20 min. A 100 mL solution of 50 mM Trizma (Tris) buffer was then prepared with DI water in a 250 mL beaker, after which the pH was adjusted to 8.50 ± 0.02 with 1 M HCl and NaOH solutions. The solution was then divided into two aliquots of 75 mL (Aliquot **A**) and 25 mL (Aliquot **B**). Substrates washed in a 3:1 piranha clean were placed in a custom-machined PTFE holder and fully submerged in **A**. 128 mg of CuSO_4_·5H_2_O (for a final concentration of 5.1 mM in the combined solution) was dissolved in **B** under vigorous stirring. 200 μL of 30% H_2_O_2_ (19.6 mM final concentration) as well as 200 mg of DA (2 mg/mL final concentration) were measured out in preparation for the next step.

The DA was rapidly dissolved in **A** under vigorous stirring. Simultaneously, H_2_O_2_ was added to **B** under vigorous stirring. After the color of **B** had completely changed (within 1–2 s), **B** was combined with **A** while maintaining vigorous stirring. The solution was left under moderate stirring for an additional 30 min. Finally, substrates were removed from the solution, thoroughly washed with DI water, dried with a stream of pressurized air, and stored for up to 10 days in the dark until used.

### 4.4 XPS Analysis

XPS was performed with a PHI VersaProbe III XPS instrument equipped with a monochromatic 50 W Al Kα (1486.6 eV) source with a 1000 μm × 200 μm analysis area and emission angle of 45^◦^. Charge control was done with a low energy electron flood and a low energy Ar^+^ flood. The graphitic carbon peak at 284.8 eV was designated as the reference for charge calibration. The regions of C 1s, O 1s, N 1s, and Cu 2p, were measured at a pass energy of 26, 26, 26, and 55 eV with a step size of 0.1, 0.1, 0.1, and 0.2 eV respectively.

### 4.5 Laser-Patterned Silver Deposition

**Figure 11:**
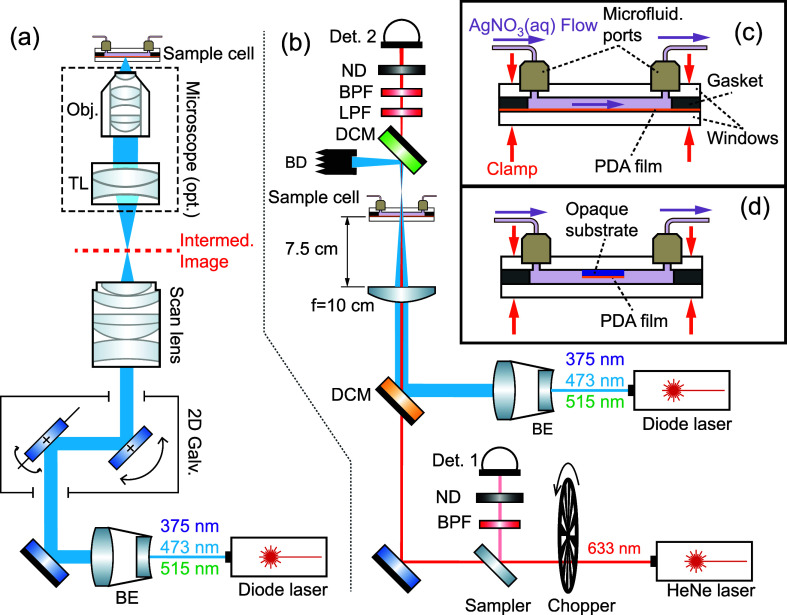
Schematics of the optical setups. (a) Laser-scanning immersion lithography setup. If the microscope is not used, the sample cell is placed at the intermediate image plane of the scan lens. (b) In situ deposition monitoring setup. (c) Optical flow cell where the PDA-coated lower window doubles as a transparent sample. (d) Optical flow cell with sample mounted onto the upper cell window.

The experiment was performed inside a microfluidic cell consisting of two glass windows clamped across one or two 0.3 mm thick ring-shaped neoprene gaskets. PDA, FO-PDA, or PNE was deposited on the lower window (Figure 11c), or on a separate piece of silicon that was mounted onto the upper window with a small amount of marine epoxy (Figure 11d).

The cell was connected to a closed, airtight liquid pumping system by way of mini luer microfluidic connectors (Fluidic 997 mini Luer Connector and Fluidic 631 Through Hole Connector from Labsmith) that allow liquid to be passed across the substrate while it is illuminated from below. Low permeability PEEK tubing was used throughout the setup to minimize the diffusion of oxygen into the circulating salt solution. A 30 mL aqueous 50 mM AgNO_3_ solution was prepared under argon, and then bubbled with argon for at least one hour. The pumping system was then repeatedly purged with argon, and the solution was injected into the pumping system’s storage bottle, from where it was circulated through the cell with a peristaltic pump at approximately 20 mL h^–1^. The circulation was maintained for at least 15–20 min before the laser was switched on. At the same time, the liquid in the storage bottle was bubbled with a slow stream of argon. Both the circulation and argon flow were maintained for the entire experiment.

Laser-scanning immersion lithography was performed with the setup illustrated in Figure 11a. A beam from a digitally modulated laser diode module at 375 nm (Vortran Stradus 375-60), 473 nm (Huebner Photonics Cobalt 06-MLD 100 mW), and 515 nm (Huebner Photonics Cobalt 06-MLD 150 mW) was enlarged in a home-built beam expander. The light was directed to a 2D small beam galvanometer mirror assembly (Thorlabs GVS002 and GCM102) that produced the lithographic pattern, and then on to a scan lens (Thorlabs LSM03-VIS), which focused the scanned pattern on the intermediate image plane. For most experiments, a home-built microscope transferred the image onto the substrate inside the liquid-filled cell via a tube lens (Thorlabs TL300-A) and a 10x microscope objective (Olympus Plan N, 10×/0.25). When high spatial resolution was not required, it was also possible to dispense with the microscope and place the sample cell directly at the scan lens’ intermediate image plane. In both cases, the lithographic pattern was produced by repeatedly scanning the galvanometer in the desired pattern, until the target exposure dose had been reached.

### 4.6 In Situ Red Light Monitoring of Deposition

To measure the Ag deposition in situ, the transparent sample cell in Figure 11c was used, but the focusing optic in this case (Figure 11b) consisted of a single convex focusing lens with a *f* = 10 cm focal length. The lens was placed  7.5 cm from the substrate, resulting in a 4-fold reduction in laser spot diameter on the substrate. When measuring thermal silver deposition, the beam was instead expanded by a factor of 3.3 by inserting a concave *f* = −3.0 cm lens (not shown) 7 cm below the convex lens, producing a Galilean beam expander. The light from the laser diodes was combined with collimated light from a red HeNe laser (Spectraphysics model 106-1, λ = 632 nm) using a dichroic mirror (Thorlabs DMLP505).

Since the red beam was not expanded, its spot diameter  0.45 mm on the substrate was approximately one  the spot diameter  1.3 mm of the depositing light, ensuring that the depositing light intensity *I*_0_ was relatively constant throughout the sampled region. More precisely, if we assume Gaussian intensity distributions in both the red and the depositing beam, the average intensity probed by the red beam was approximately 90% of the peak intensity of the blue light:
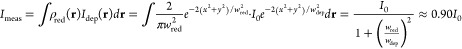
2where  is the intensity profile of the red laser on the substrate, normalized so that its integral equals 1. In all dose calculations in this paper, the value of  was used, where *P* is the total optical power incident on the sample.

The shorter wavelengths were removed from the light that passed through the substrate with several filters: A long-pass dichroic mirror, a neutral density filter, a long pass filter (Thorlabs DMLP505, NE06B, and FGL630M), and a bandpass filter (Andover Corp 630FS10). The light was then measured with a Si photodetector (Thorlabs DET36A). A small percentage of the red light was also diverted with a beam sampler (Thorlabs BSF10-A) before reaching the sample and was filtered and detected in a similar manner. The diverted beam’s signal was used to normalize the output of the first detector to compensate for power fluctuations and drift in the red laser.

## Supporting Information Available

Green light exposure graph of extinction vs time; graphs of patterned samples as a function of time since fabrication; discussion of dose calculation for ISO # 2 test pattern analysis; discussion of Rayleigh resolution limit for test patterns; discussion of resolution analysis of test pattern SEM micrographs; graphed estimation of percolation threshold (D′)_perc_ in PDA, PNE, and FO-PDA films; Overall XPS spectra for PDA, PNE, FO-PDA, and FO-PDA 2ox films; fitting curves of the copper spectra for FO-PDA, and FO-PDA 2ox films; detailed schemes for PDA and PNE formation; and SEM images of silver deposition on PDA, PNE, and FO-PDA films at low optical doses (PDF)
