# Supplementary material for: Microscale Metal Patterning on Any Substrate: Exploring the Potential of Poly(dopamine) Films in High Resolution, High Contrast, Conformal Lithography
Source: ACS Appl Mater Interfaces. 2024 Nov 20;16(48):66387–401. doi: 10.1021/acsami.4c07115 (PMC11622185; doi:10.1021/acsami.4c07115)
Supplement: Supplementary file 1 — am4c07115_si_001.pdf [file am4c07115_si_001.pdf]

Supporting Information

Microscale Metal Patterning on Any Substrate: Exploring the Potential of Poly(dopamine) Films in High Resolution, High Contrast, Conformal Lithography

Elliott D. Kunkel, C. Blake Loker, Hunter N. Cowden, Hans D. Robinson*

Department of Physics, Virginia Tech, Blacksburg, Virginia 24060

* E-mail: hansr@vt.edu

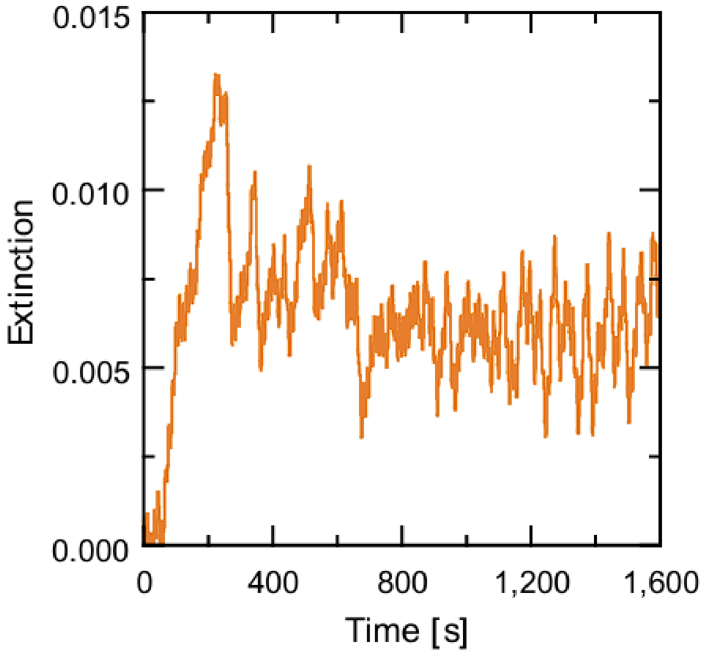

Figure S1: Green light exposure on PDA film producing no silver deposition at an intensity of 30 W/cm^2

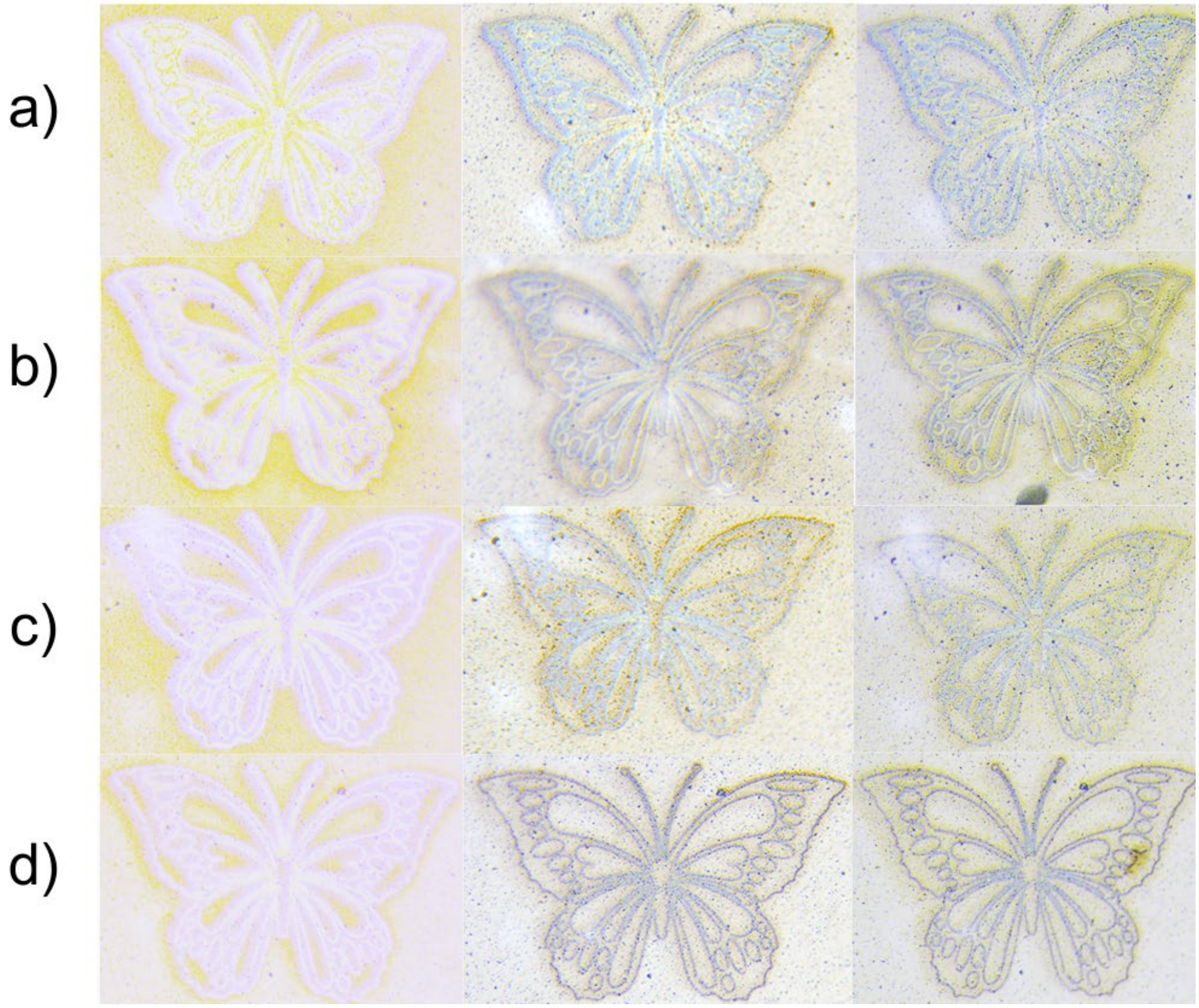

Figure S2: Evolution of samples exposed with different powers over the course of 2 weeks. Left column: micrographs of silver patterns taken immediately after exposure. Middle column: 5 days after exposure. Right column: 10 days after exposure. Exposure doses vary by row: a) 0.59 J/mm, b) 0.29 J/mm, c) 0.19 J/mm, d) 0.15 J/mm

Discussion S1. Dose calculations

These formulae (in addition to **Eq. 2** in the main paper) were used to convert between the different measures of light intensity, power, line dose and area dose. We approximate the intensity distribution of the laser beam on the substrate with a Gaussian:

$$I(x,y) = I_0 e^{-2\frac{x^2+y^2}{w^2}} \qquad (\boldsymbol{S1})$$

where I_0 is the peak intensity of the beam. The integral of $I(x,y)$ equals the total power of the laser beam:

$$P = \int I(x,y)dxdy = \frac{1}{2}\pi w^2 \cdot I_0. \qquad (\boldsymbol{S2})$$

As the galvanometer scans the laser focus across the substrate at speed v, the line dose (in J/mm) equals:

$$D_l = \frac{P}{v} \qquad (\boldsymbol{S3})$$

The area dose at the center of scanned line is:

$$D_a = \frac{D_l}{\frac{1}{I_0}\int I(x,0)dx} = \frac{D_l}{\sqrt{\frac{\pi}{2}}w} \qquad (\boldsymbol{S4})$$

Since this maximum dose is only provided along a line, the doses in **Fig. 3-5, 9** in the main paper are given as 90% of this value (by **Eq. 2** of main paper). If multiple lines at a line density of σ lp/mm (line pairs/mm) are drawn parallel to each other, and sufficiently close that individual lines cannot be resolved, the average area dose is instead

$$D_a = D_l \cdot \sigma = \frac{D_r}{r} \qquad (\boldsymbol{S5})$$

where r is the line spacing of the pattern.

Discussion S2. Resolution analysis

We quantitatively analyze the resolution from the SEM micrographs in **Figure 2** and **Figure 3** in the main paper by integrating the brightness along the horizontal and vertical lines and comparing with a Gaussian approximation of a diffraction pattern at the far-field limit. The Guassians were fit to the Airy disks in this pattern by requiring the integral of the two distributions to be equal. This leads to a Gaussian $1/e^2$ beam radius

$$w = \frac{2\sqrt{2}}{j_{1,1}} r_R = 0.74 r_R \qquad (\boldsymbol{S6})$$

where $j_{1,1}$ is the first positive zero of the first Bessel function of the first kind, and r_R is the Rayleigh resolution limit. For the estimated $r_R = 1.7\ \mu\text{m}$ in the paper, this gives $w = 1.25\ \mu\text{m}$, which is the valued used in the dose calculations described above.

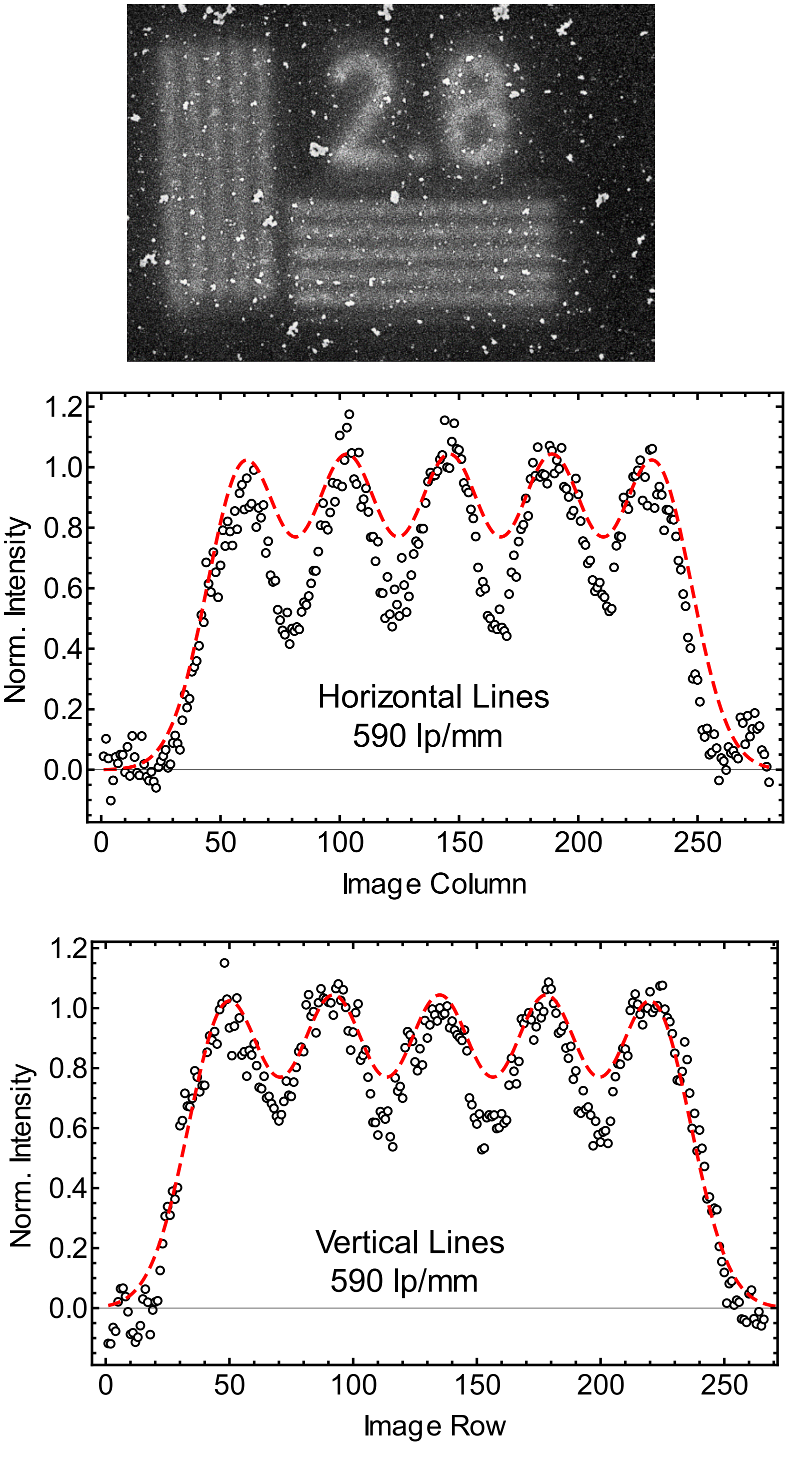

Figure S3: Summation along vertical and horizontal lines in 590 lp/mm (1.7 μm resolution) pattern at dose $D = 0.12$ J/mm, rotated to ensure line alignment with axes. Dashed red lines indicate Rayleigh diffraction limit.

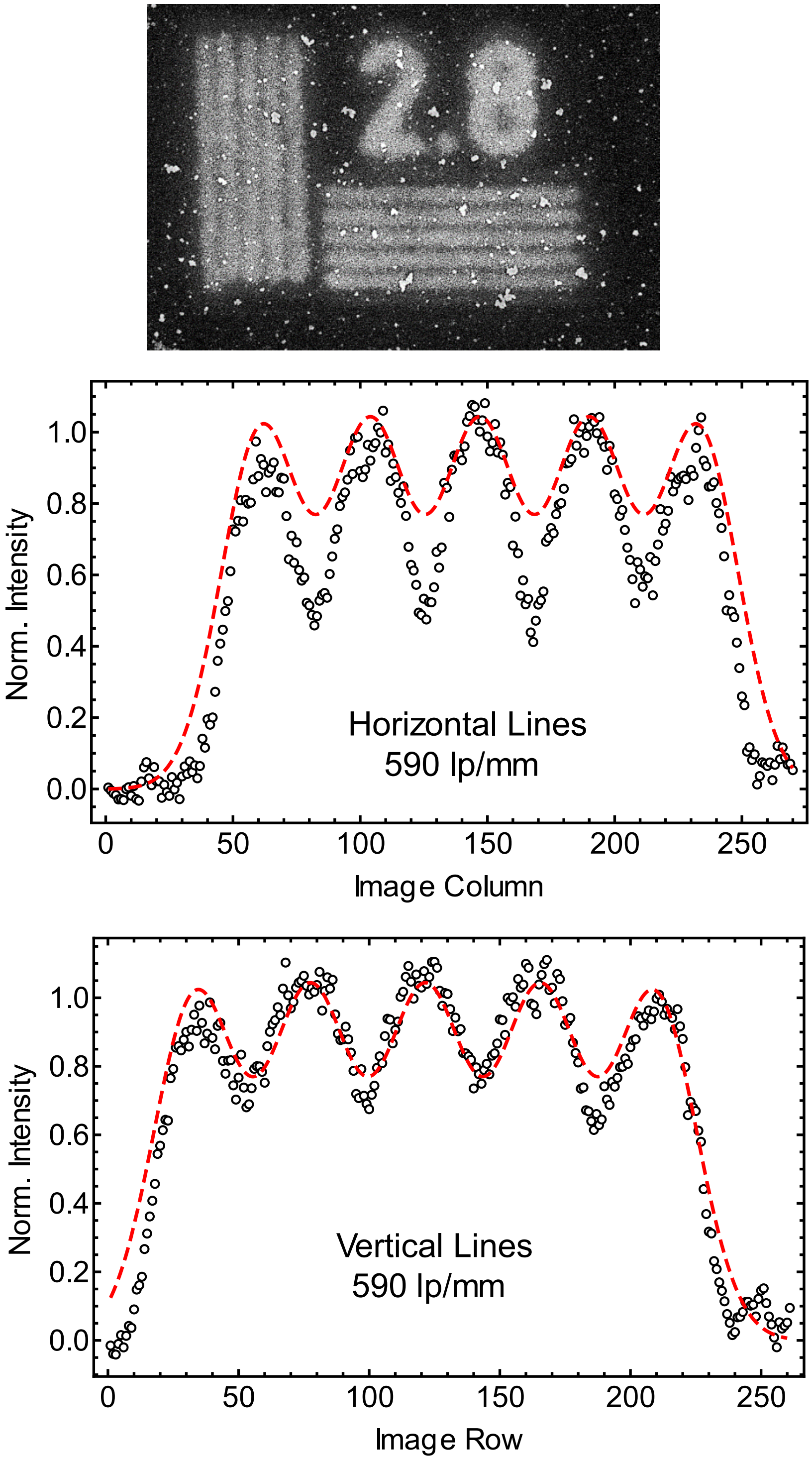

Figure S4: Summation along vertical and horizontal lines in 590 lp/mm (1.7 μm resolution) pattern at dose $D = 0.25$ J/mm, rotated to ensure line alignment with axes. Dashed red lines indicate Rayleigh diffraction limit.

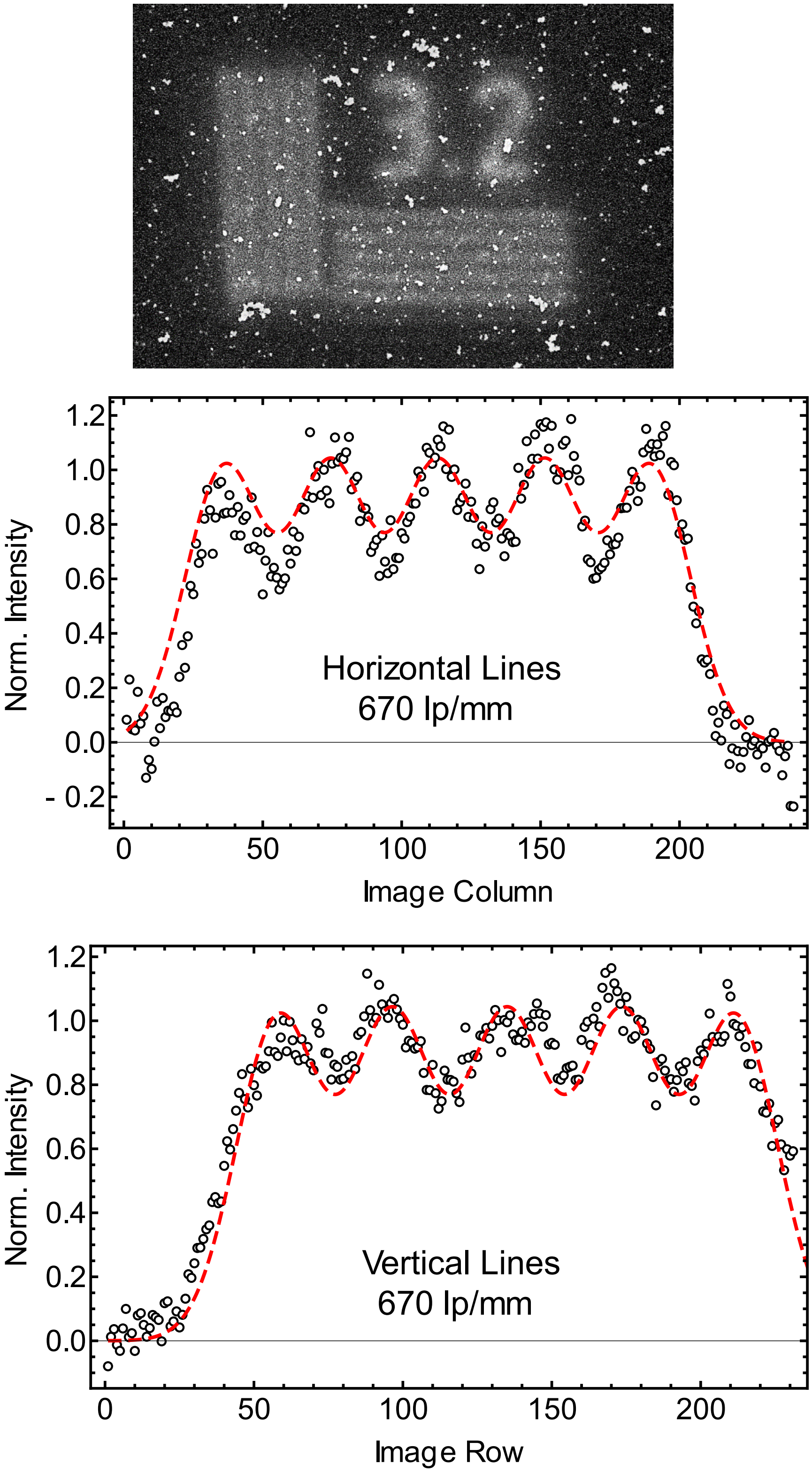

Figure S5: Summation along vertical and horizontal lines in 670 lp/mm (1.5 μm resolution) pattern at dose $D = 0.12$ J/mm, rotated to ensure line alignment with axes. Dashed red lines indicate Rayleigh diffraction limit.

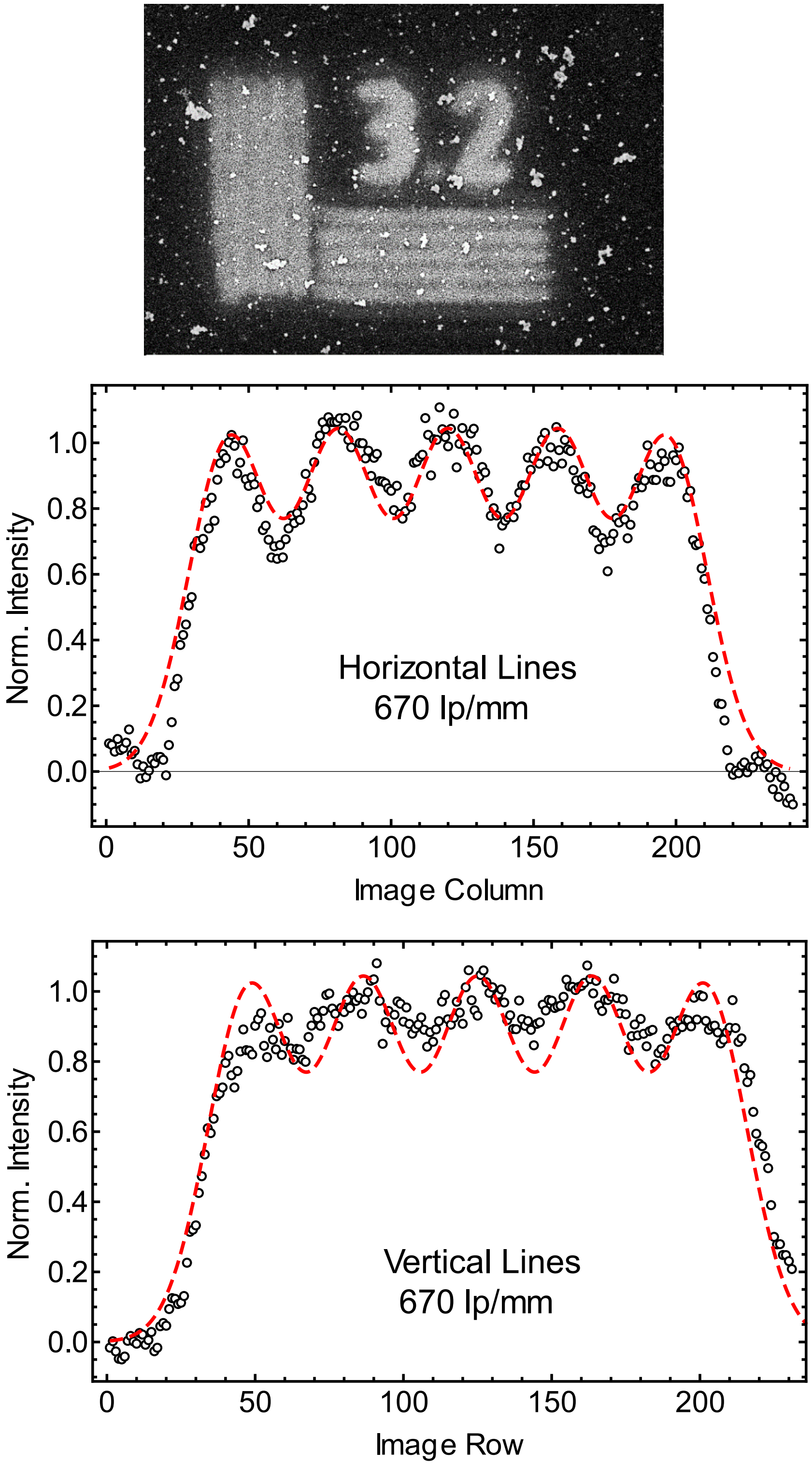

Figure S6: Summation along vertical and horizontal lines in 670 lp/mm (1.5 μm resolution) pattern at dose $D = 0.25\ \mathrm{J/mm}$, rotated to ensure line alignment with axes. Dashed red lines indicate Rayleigh diffraction limit.

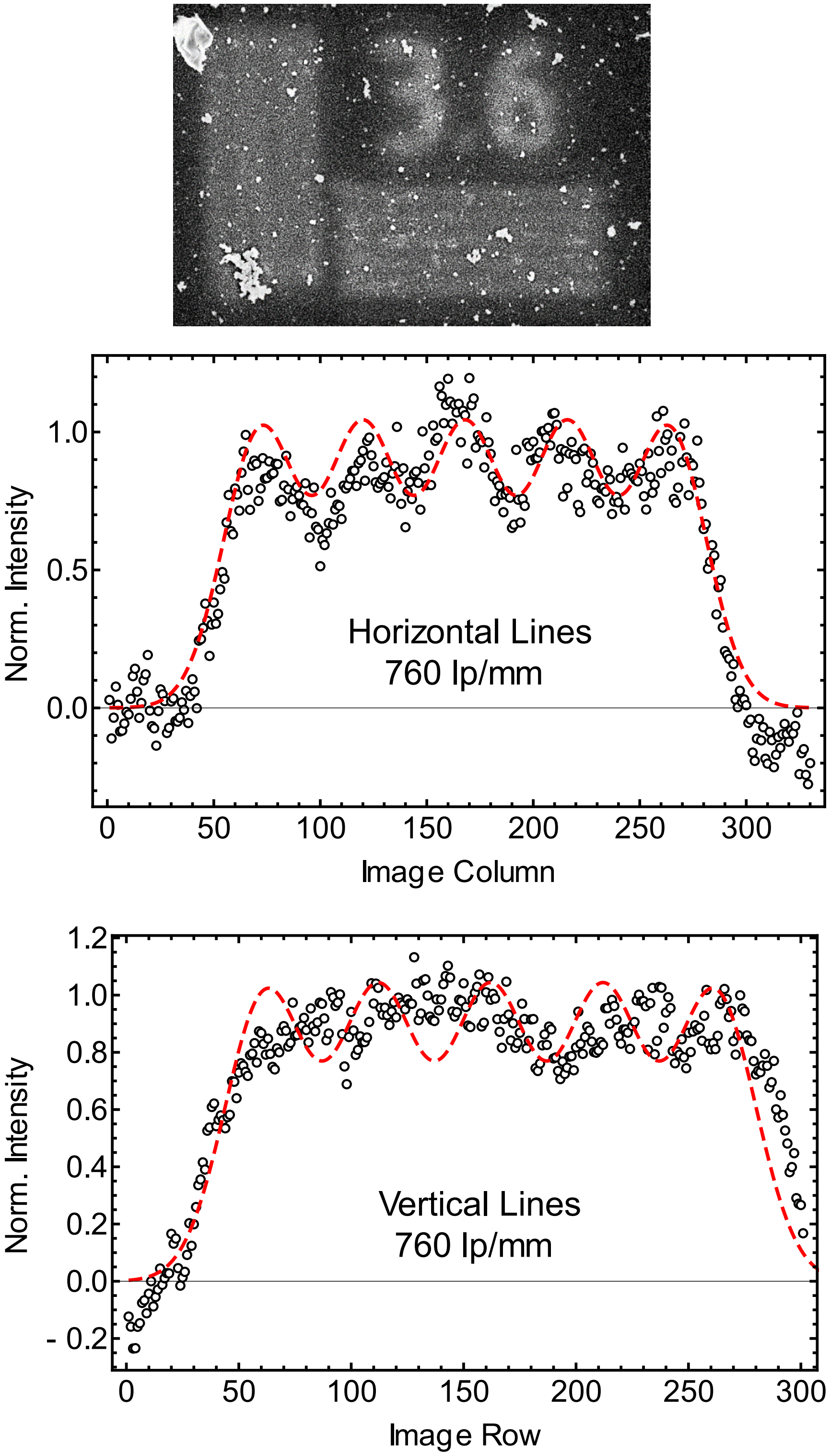

Figure S7: Summation along vertical and horizontal lines in 760 lp/mm (1.3 μm resolution) pattern at dose $D = 0.12$ J/mm, rotated to ensure line alignment with axes. Dashed red lines indicate Rayleigh diffraction limit.

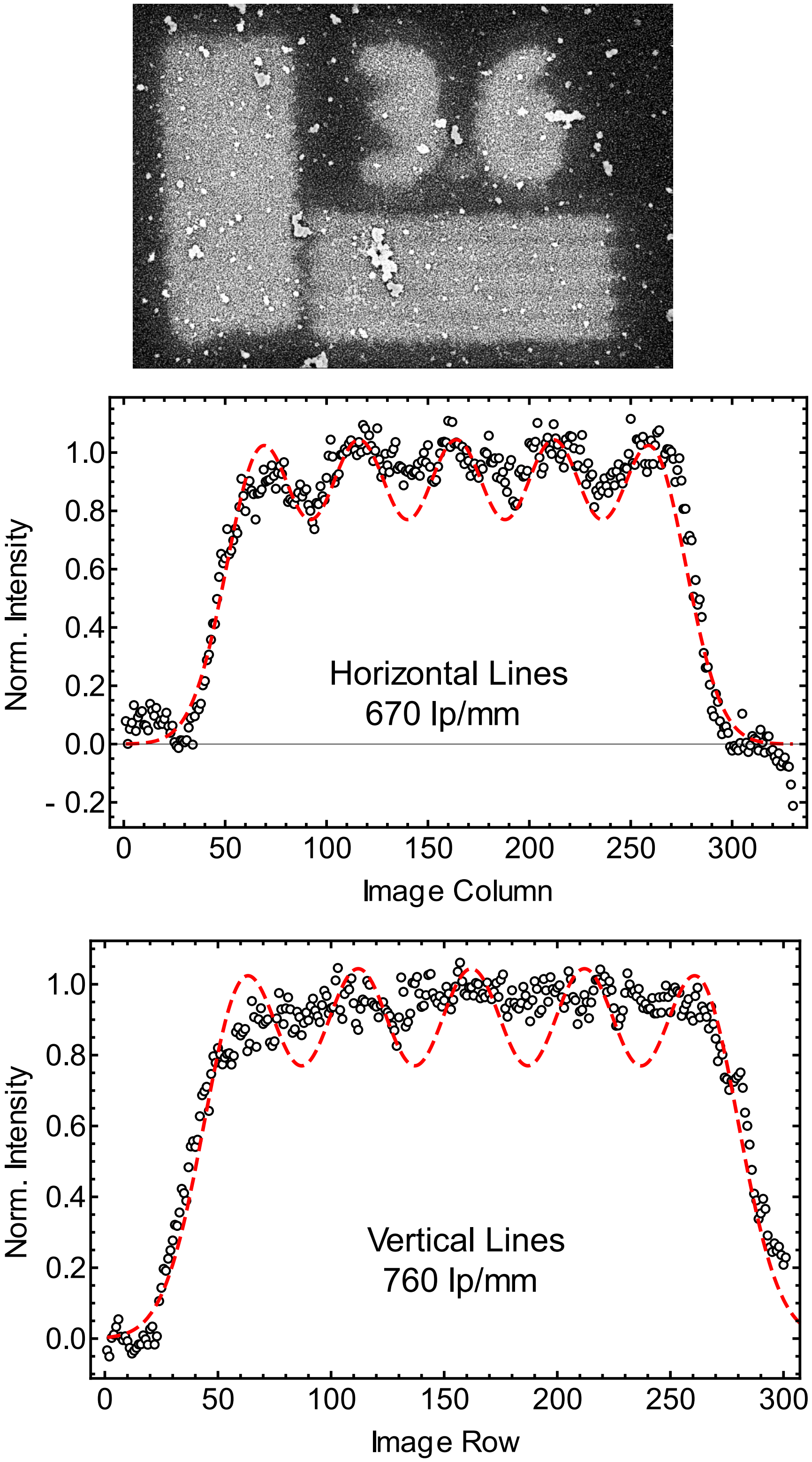

Figure S8: Summation along vertical and horizontal lines in 760 lp/mm (1.3 μm resolution) pattern at dose $D = 0.25$ J/mm, rotated to ensure line alignment with axes. Dashed red lines indicate Rayleigh diffraction limit.

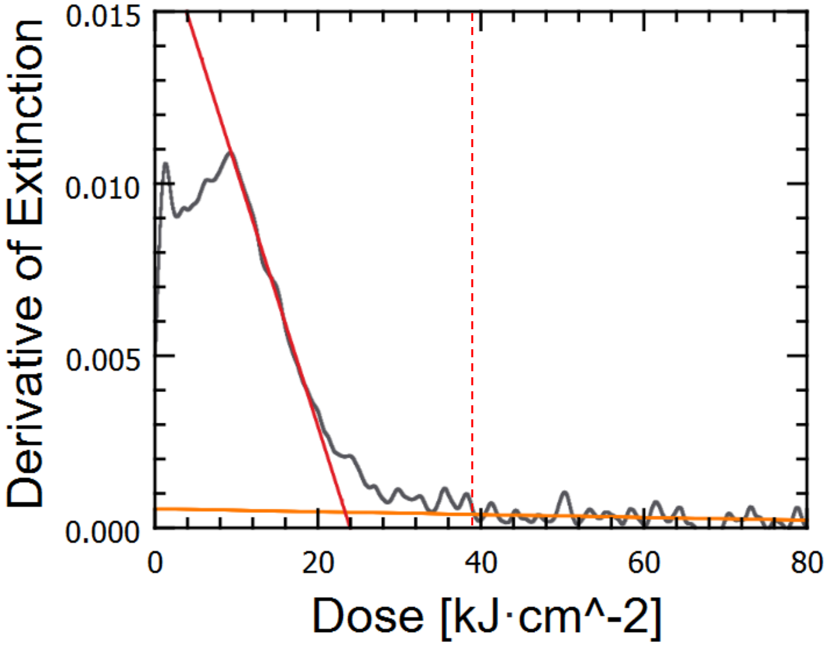

Figure S9: Smoothed numerical derivative of PDA data from **Fig. 10(a)**. The second derivative $E''(D)$ of the extinction initially has a large negative value (the slope of the solid red line), corresponding to island growth in the film. It then transitions to a much smaller value (slope of the black line) that we associate with the growth of a continuous metal film. The estimate D'_{perc} of the optical percolation transition is taken to equal the dose at the end of the transition to the lower value of $E''(D)$ (dashed red line).

.

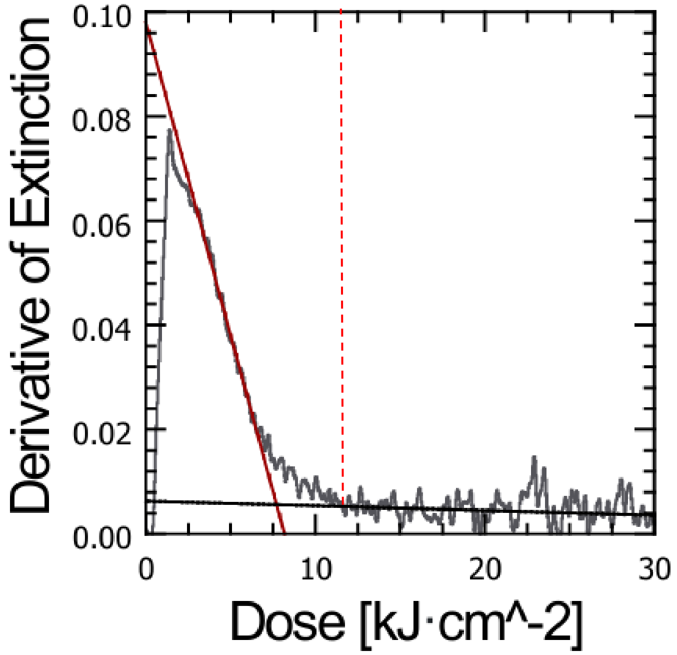

Figure S10: Smoothed numerical derivative of PNE data from **Fig. 10(b)**. The second derivative $E''(D)$ of the extinction initially has a large negative value (the slope of the solid red line), corresponding to island growth in the film. It then transitions to a much smaller value (slope of the black line) that we associate with the growth of a continuous metal film. The estimate D'_{perc} of the optical percolation transition is taken to equal the dose at the end of the transition to the lower value of $E''(D)$ (dashed red line).

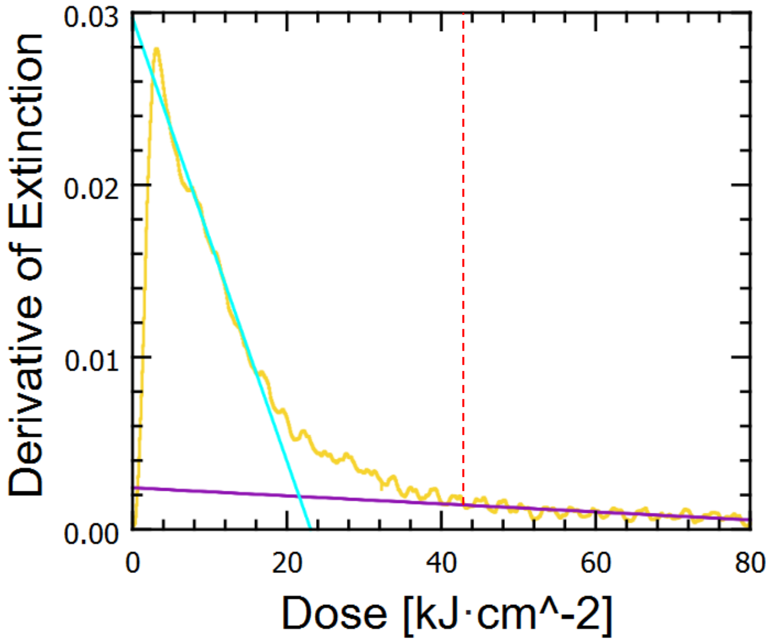

Figure S11: Smoothed numerical derivative of FO-PDA data from **Fig. 10(c)**. The second derivative $E''(D)$ of the extinction initially has a large negative value (the slope of the solid red line), corresponding to island growth in the film. It then transitions to a much smaller value (slope of the black line) that we associate with the growth of a continuous metal film. The estimate D'_{perc} of the optical percolation transition is taken to equal the dose at the end of the transition to the lower value of $E''(D)$ (dashed red line).

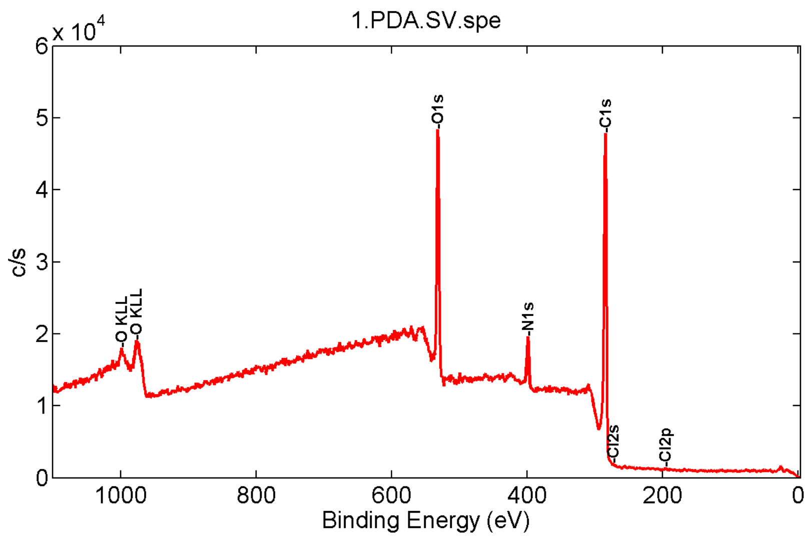

Figure S12: PDA XPS Spectra

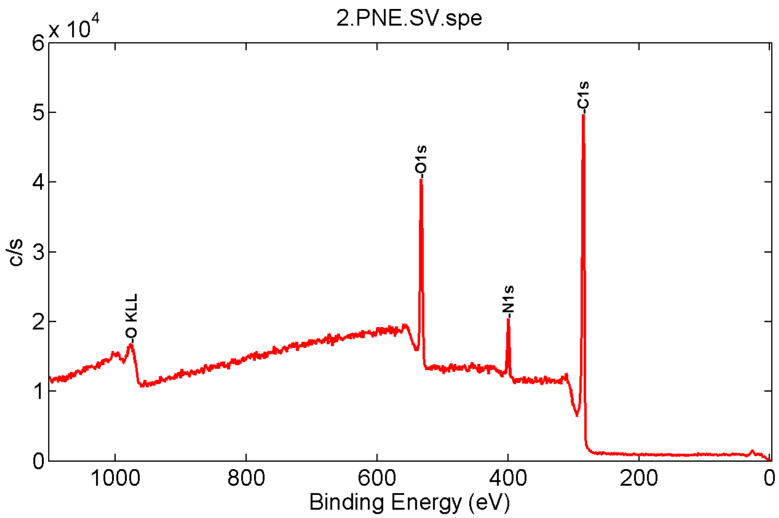

Figure S13: PNE XPS Spectra

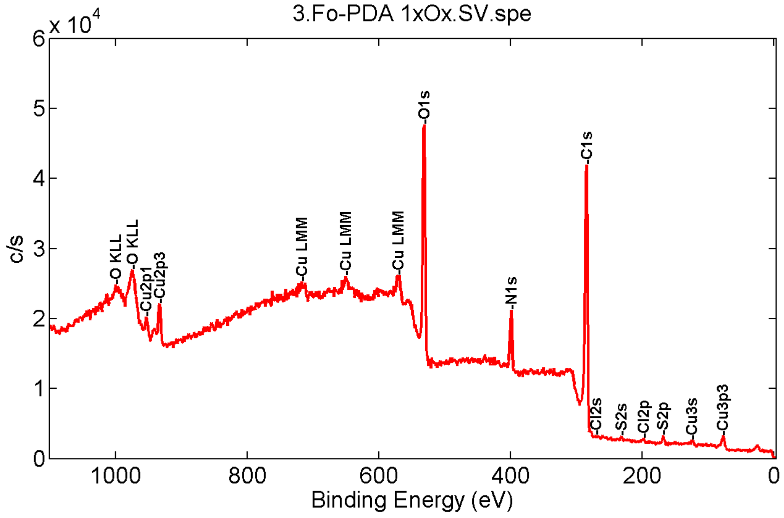

Figure S14: FO-PDA 1xOx XPS Spectra

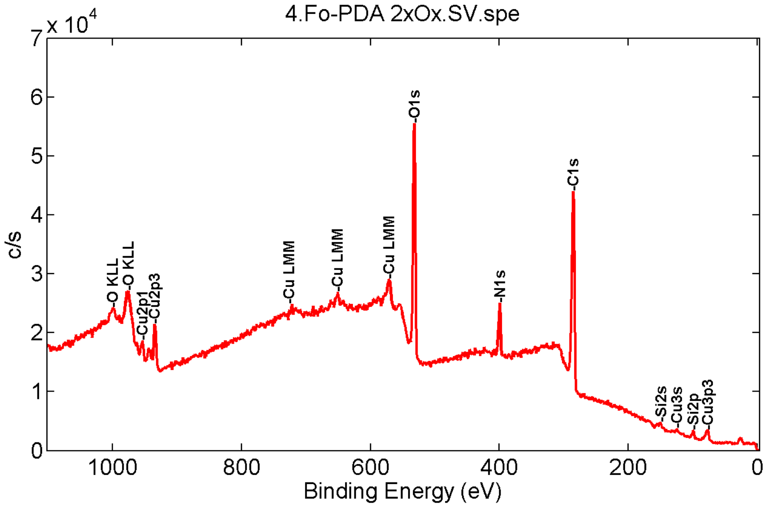

Figure S15: FO-PDA 2xOx XPS Spectra

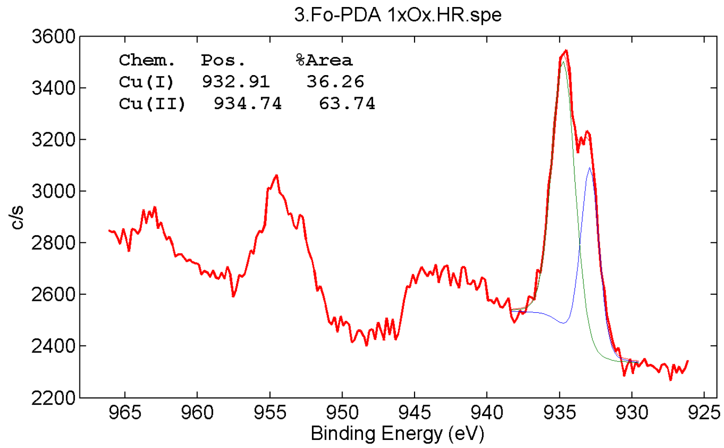

Figure S16: FO-PDA 1xOx Cu Spectra displaying the relative atomic percentage of Cu(I) and Cu(II).

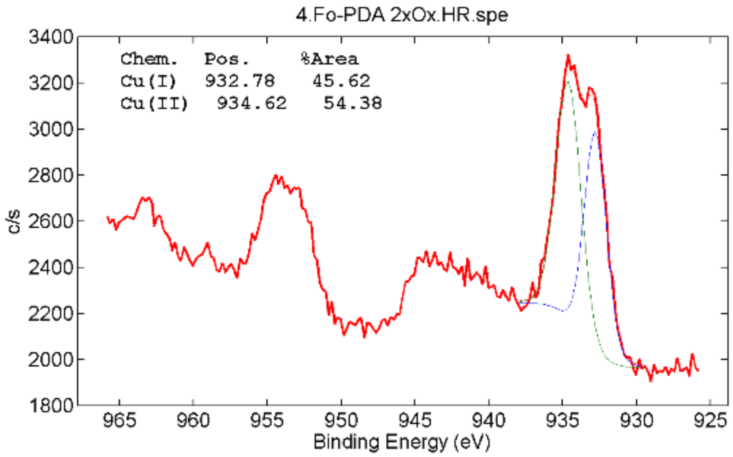

Figure S17: FO-PDA 2xOx Cu Spectra displaying the relative atomic percentage of Cu(I) and Cu(II).

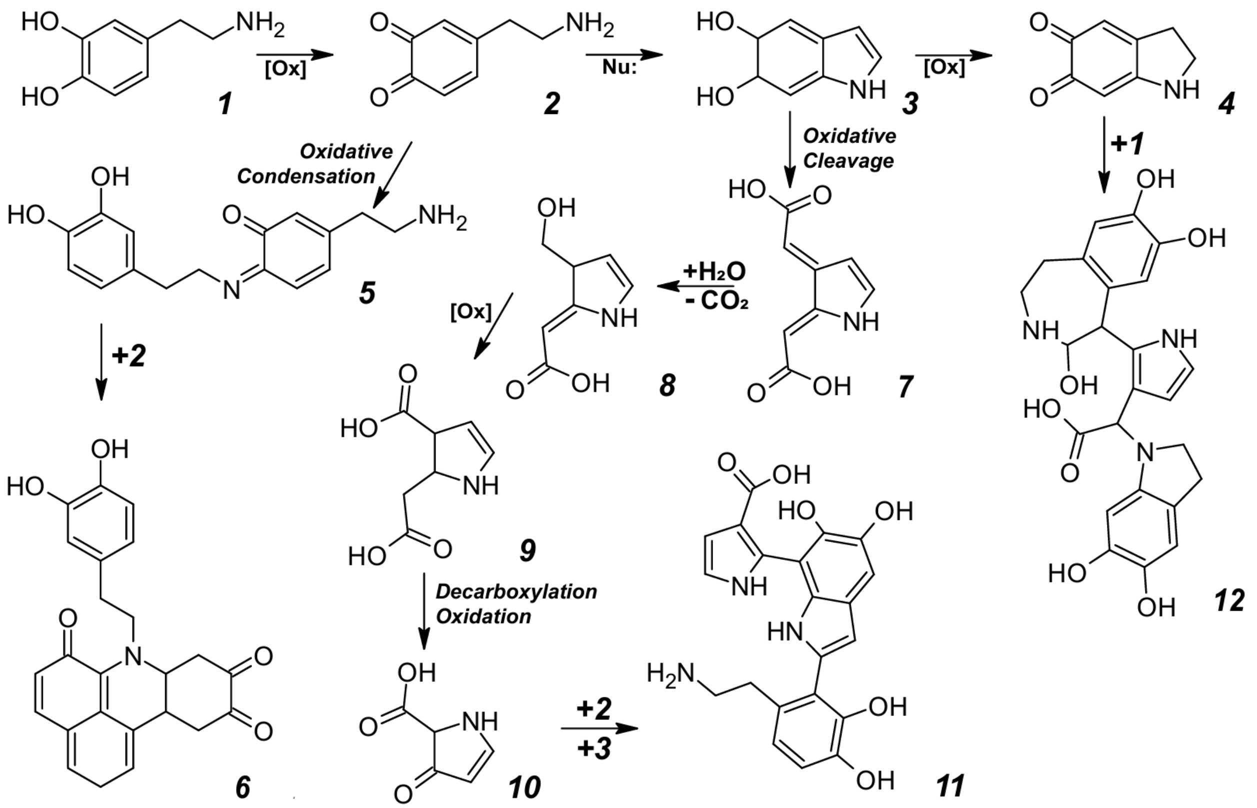

Scheme S1: Example of PDA reaction pathways that are mentioned in literature. 1-4 is known as the general pathway for the PDA reaction.[8,7]

1. Dopamine (DA)
2. Dopamine-O-quinone (DA quinone)
3. 5,6-Dihydroxyindoline (DHI)
4. Dopaminochrome (DAC)

5-6. Oxidative condensation of the DA quinone creates **5**, which then reacts with **2** again to make porphyrin-type tetramers.[7]

7-11. Oxidative cleavage of DHI units leads to pyrrole-2,3-dicarboxylic acid (PDCA) **(9)** and pyrrolecarboxylic acid (PCA) **(10)** units that are found in mass spectrometry.[7,8]

4,12. Pathway for trimer based structure found of (DHI)/PCA complexes using mass spec and that have been proposed to dominate the beginning of the reaction using time-of-flight secondary ion mass spectrometry (ToF-SIMS), and matrix-assisted laser desorption/ionization mass spectrometry.[8]

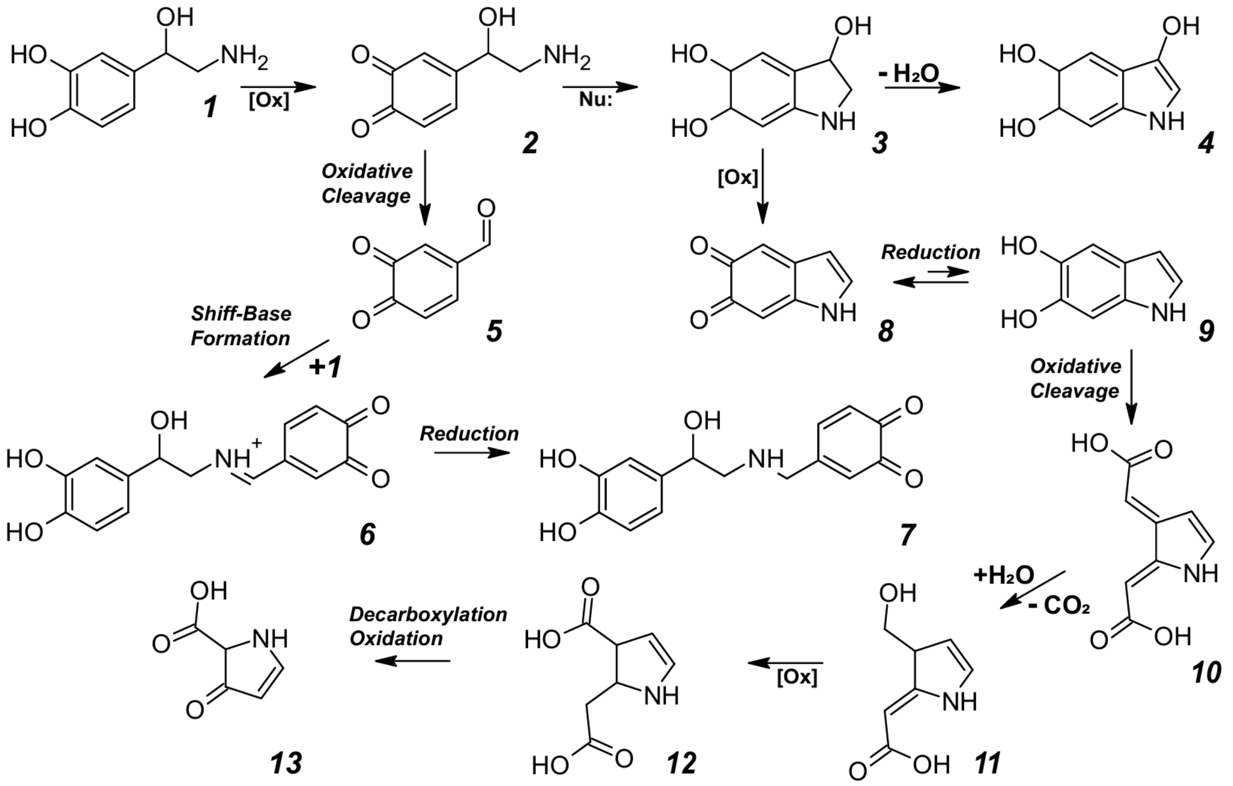

Scheme S2: Example of PNE reaction pathways that are mentioned in literature. **1 - 4** is known as the general pathway for the PNE reaction.[2]

1. Norepinephrine (NE)
2. Norepinephrine-o-quinone
3. Leuconoradrenochrome
4. Noradrenolutin
5. 3,4-dihydroxybenzaldehyde (DHBA)

6-7. Shiff-Base reduction of DHBA reacting with O-quinone.[2,3]

8-13. Reducing to **9** is not a favorable reaction; however, a pH of 8.5 wouldn't be high enough to stop reduction reactions occurring completely, which would lead to the same reaction occurring in both PNE formation and PDA formation. Similar compounds have also been reported in literature as a result of oxidative cleavage resulting in other carboxylates.[5,6]

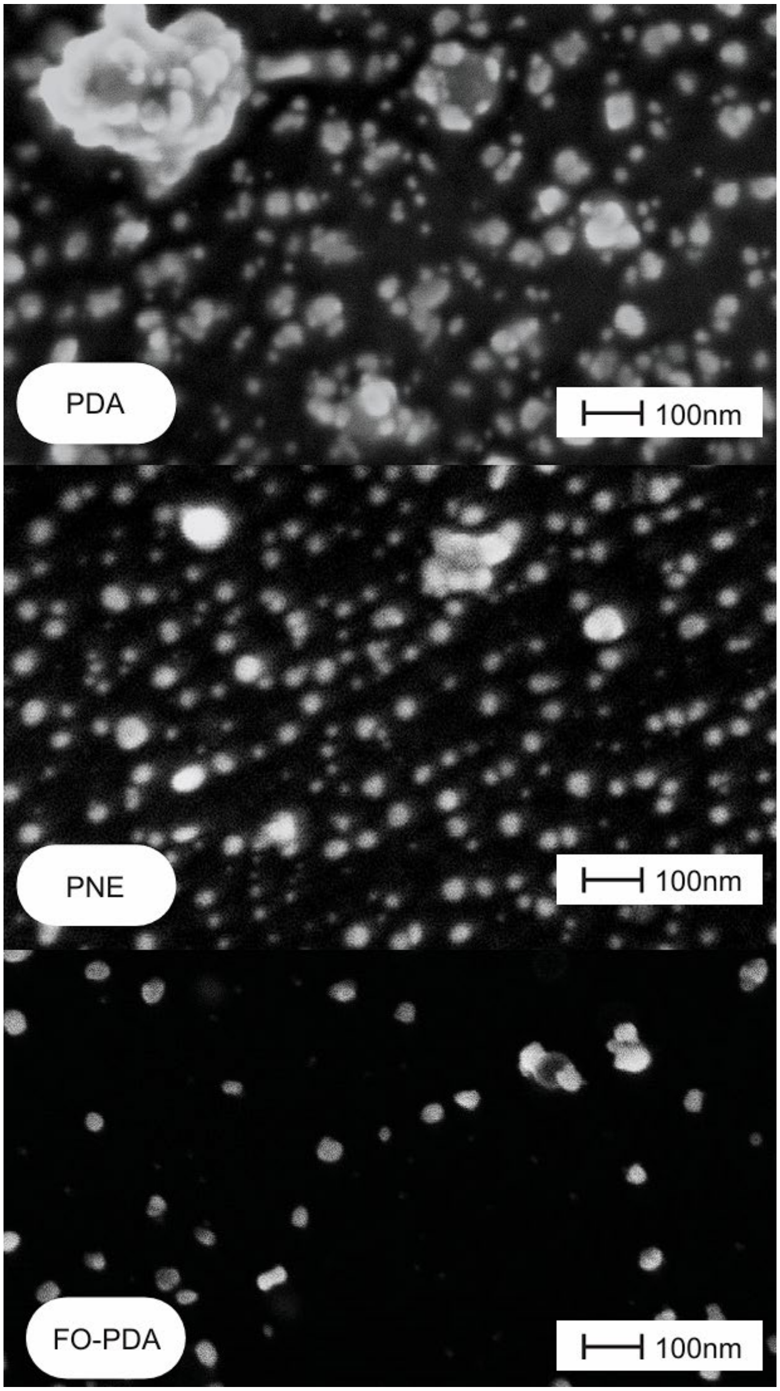

Figure S18: SEM images of silver deposition on PDA, PNE, and FO-PDA films at low optical doses.

References

1. Kang, S. M.; Rho, J.; Choi, I. S.; Messersmith, P. B.; Lee, H. Norepinephrine: Material-Independent, Multifunctional Surface Modification Reagent. *J. Am. Chem. Soc.* **2009**, *131* (37), 13224–13225. DOI: 10.1021/ja905183k.
2. Hong, S.; Kim, J.; Na, Y. S.; Park, J.; Kim, S.; Singha, K.; Im, G. I.; Han, D. K.; Kim, W. J.; Lee, H. Poly(norepinephrine): Ultrasmooth Material-Independent Surface Chemistry and Nanodepot for Nitric Oxide. *Angew. Chem. Int. Ed.* **2013**, *52* (35), 9187–9191. DOI: 10.1002/anie.201301646.
3. Lu, Z.; Teo, B. M.; Tabor, R. F. Recent Developments in Polynorepinephrine: An Innovative Material for Bioinspired Coatings and Colloids. *J. Mater. Chem. B* **2022**, *10* (39), 7895–7904. DOI: 10.1039/D2TB01335E.
4. Cho, J. H.; Katsumata, R.; Zhou, S. X.; Kim, C. B.; Dulaney, A. R.; Janes, D. W.; Ellison, C. J. Ultrasmooth Polydopamine Modified Surfaces for Block Copolymer Nanopatterning on Flexible Substrates. *ACS Appl. Mater. Interfaces* **2016**, *8* (11), 7456–7463. DOI: 10.1021/acsami.6b00626.
5. Schüsler-Van Hees, M. T. I. W.; Beijersbergen Van Henegouwen, G. M. J.; Stoutenberg, P. Autoxidation of Catechol(amine)s. *Pharm. Weekbl.* **1985**, *7* (6), 245–251. DOI: 10.1007/BF01959197.
6. Manini, P.; Panzella, L.; Napolitano, A.; d'Ischia, M. Oxidation Chemistry of Norepinephrine: Partitioning of the O-Quinone between Competing Cyclization and Chain Breakdown Pathways and Their Roles in Melanin Formation. *Chem. Res. Toxicol.* **2007**, *20* (10), 1549–1555. DOI: 10.1021/tx700254q.
7. Alfieri, M. L.; Micillo, R.; Panzella, L.; Crescenzi, O.; Oscurato, S. L.; Maddalena, P.; Napolitano, A.; Ball, V.; d'Ischia, M. Structural Basis of Polydopamine Film Formation: Probing 5,6-Dihydroxyindole-Based Eumelanin Type Units and the Porphyrin Issue. *ACS Appl. Mater. Interfaces* **2018**, *10* (9), 7670–7680. DOI: 10.1021/acsami.7b09662.
8. Ding, Y.; Weng, L.-T.; Yang, M.; Yang, Z.; Lu, X.; Huang, N.; Leng, Y. Insights into the Aggregation/Deposition and Structure of a Polydopamine Film. *Langmuir* **2014**, *30* (41), 12258–12269. DOI: 10.1021/la5026608.
